# Designing Culturally Acceptable, Nutritious, and Low Environmental Impact Finnish Diets with Mycoprotein: A Novel Optimization Approach

**DOI:** 10.1016/j.cdnut.2025.107559

**Published:** 2025-09-22

**Authors:** Yue Fu, Xavier Irz

**Affiliations:** Department of Economics and Management, University of Helsinki, Helsinki, Finland

**Keywords:** diet optimization, sustainable diet, mycoprotein, meat alternatives, environmental impact

## Abstract

**Background:**

Meat alternatives can support a gradual reduction in meat consumption, contributing to more sustainable diets. Optimization is a useful tool to investigate this potential, but neglecting diet acceptability may limit the relevance of the approach.

**Objectives:**

The aim of this study was to develop a new diet optimization model that includes meat alternatives and accounts for diet acceptability, then to apply it to characterize a culturally acceptable, nutritionally adequate, and low-climate impact Finnish diet that incorporates mycoprotein.

**Methods:**

An original quadratic optimization model minimizing the weighted distance from the current diet was designed, with mycoprotein introduced as a new food within the meat group. The model first simulated a nutritionally adequate diet including mycoprotein (“NUTR” diet) and then progressively reduced dietary greenhouse gas emissions (GHGEs) while maintaining total consumption of the meat group. Model performance was assessed against a piecewise linear model, and robustness was tested through a sensitivity analysis.

**Results:**

The meat group in the “NUTR” diet, which included 27.3 g (5.9 g) of mycoprotein, amounted to 82% (87%) of observed meat consumption for an average adult male (female). For further GHGE reductions, red and processed meats were replaced mainly by poultry, followed by mycoprotein with an increasing share. Imposing large reductions in GHGEs while maintaining meat consumption to its “NUTR” level led to larger adjustments in other food groups and raised potential nutritional concerns, whereas these issues were less pronounced for small to moderate GHGE reductions. Compared with the piecewise linear model, the quadratic formulation was less sensitive to baseline assumptions, yielding more robust and realistic diets.

**Conclusions:**

The proposed model advances diet optimization for the study of new foods by incorporating improved considerations of diet acceptability. The Finnish case study illustrates its applicability to simulate climate-friendly, nutritionally adequate diets that integrate mycoprotein, offering a valuable tool for future research on novel foods.

## Introduction

As a key strategy to reduce greenhouse gas emissions (GHGEs) from the food system [1], the search for sustainable diets—defined as “diets with low environmental impacts which contribute to food and nutrition security and to healthy life for present and future generations” [2]—has received increasing attention. However, achieving sustainable dietary change remains challenging, particularly with regard to reductions in meat consumption commonly advocated in Western diets [3]. Indeed, global meat demand is projected to continue growing [4], whereas important psychological barriers and cultural norms have been shown to hinder change [5]. In response, novel meat alternatives (thereafter called “meat alternatives” or “new foods”), which are designed to closely resemble conventional meat [6], have been developed and have garnered significant interest, as they may facilitate reductions in meat consumption. Substituting meat with these alternatives could greatly decrease environmental impacts [7,8] and enhance the resilience of regional protein supply amid global trade disruptions [9]. In Europe, meat alternatives are emerging as a dietary option among consumers. According to the Smart Protein Project [10] report, 24% of surveyed European consumers consumed plant-based meats weekly, and 34% intended to purchase or consume such alternatives in the future.

Despite their promises, meat alternatives have raised public concerns because of their heterogeneous nutritional quality and health impacts [11]. Their environmental impacts often depend on the local energy mix [12] and remain uncertain, leading to doubts about their role in sustainable diets. Thus, additional analysis and evidence are needed to lift uncertainties and allow food consumers and policymakers to make informed decisions about these new dietary options. In some recent studies, optimization models have been used in pursuit of that goal. Unlike impact evaluation studies that assume patterns of substitutions among foods and assess their dietary effects [[13], [14], [15]], diet optimization takes a whole dietary approach, identifying combinations of meat alternatives and other foods that simultaneously meet different sustainability criteria. Current applications have investigated the nutritional leveling effects [16] or environmental impact mitigation potential [8,17] of including meat alternatives/novel foods into existing diets, providing quantitative insights into their role in achieving sustainable diets.

However, these diet optimization studies have not adequately addressed consumer acceptability of meat alternatives. They implicitly or explicitly assume that meat alternatives are perfect substitutes for meat by, for example, adding new foods within the meat group to allow unrestricted substitutions [16] or by modeling dietary scenarios in which all meat is replaced by alternative foods [8,17]. Although the results are useful in illustrating the maximum potential benefits of including new foods in diets, they fail to reflect the reality that consumer acceptance of such foods remains low to moderate [18]. This is a significant limitation, as the simulated dietary changes overestimate the potential of new foods to contribute to sustainable diets, potentially raising unrealistic expectations. Hence, to improve the realism and practical value of optimized diets, it is necessary to more explicitly incorporate acceptability considerations when introducing new foods.

However, this is difficult to achieve in practice for technical and other reasons. For example, the best practice in diet optimization to account for cultural acceptability is to set an objective function that minimizes deviation from the current diet. This function can be linear or quadratic, and deviations can be measured in relative or absolute terms [19]. Nevertheless, not all functional forms are appropriate when studying new foods in diets. For example, because new foods are absent in dietary surveys that define the current diet, their initial intake is 0, making it impossible to calculate relative (percentage) deviations as it would involve a division by zero [20]. In addition, piecewise linear forms fail to penalize large changes in the consumption of individual foods, often resulting in unrealistically high inclusion of new foods [21], when their nutritional or environmental properties help satisfy the constraints of the model. Finally, to recognize that meat alternatives and existing meats remain imperfect substitutes, additional penalties must be applied to the increase of the consumption of meat alternatives. Therefore, there is a need to improve existing diet optimization models to better accommodate the inclusion of new foods. Although we acknowledge a preliminary attempt to do so by Salomé et al. [16], to our knowledge, no study to date has appropriately investigated the acceptability of meat alternatives in diet optimizations by proposing and testing an improved methodology.

To fill this research gap, we integrated various approaches from the literature to develop a novel optimization model tailored to simulate diets with meat alternatives while more effectively accounting for diet acceptability—particularly by recognizing the imperfect substitutability between meats and their alternatives. Although the model is sufficiently general that it can be adapted to study any emerging meat alternative, we demonstrate its applicability by characterizing sustainable diets that incorporate mycoprotein in a Finnish context. This illustrative example was chosen for 2 reasons: first, mycoprotein is a particularly promising meat alternative because of its unique functional properties and fibrous structure [22]. Second, according to the Finnish food-based dietary guideline [23], average meat consumption in Finland should be significantly reduced for both health and environmental reasons, and large-scale production of mycoprotein is feasible in the country [24]. More specifically, we addressed the following research questions: *1*) What would be the composition of a culturally acceptable, nutritious, and low-climate impact Finnish diet that includes mycoprotein? and *2*) What are the nutritional, environmental, and cost implications of these optimized diets?

## Methods

### The diet optimization model

#### The objective function

The objective function proxies the inconvenience of dietary change by minimizing the sum of the weighted deviations from the current diet. It is expressed as follows:

(1) $$\mathrm{Min}\;\sum_{i=1}^{n}w_{i}{\left(\frac{q_{i}-q_{i}^{0}}{\sigma_{i}}\right)}^{2}+\sum_{j=1}^{m}z_{j}{\left(\frac{Q_{j}-Q_{j}^{0}}{\sigma_{j}}\right)}^{2}$$

$$=\sum_{i=1}^{n}\left(w_{i}^{+}{\left(\frac{q_{i}^{+}-q_{i}^{0}}{\sigma_{i}}\right)}^{2}+w_{i}^{-}{\left(\frac{q_{i}^{-}-q_{i}^{0}}{\sigma_{i}}\right)}^{2}\right)+\sum_{j=1}^{m}\left(2*{\left(\frac{Q_{j}^{+}-Q_{j}^{0}}{\sigma_{j}}\right)}^{2}+2*{\left(\frac{Q_{j}^{-}-Q_{j}^{0}}{\sigma_{j}}\right)}^{2}\right)$$

where

(2) $$q_{i}=q_{i}^{+}+q_{i}^{-}-q_{i}^{0}$$

(3) $$Q_{j}=Q_{j}^{+}+Q_{j}^{-}-Q_{j}^{0}$$

(4) $$q_{i}^{+}-q_{i}^{0}\geq 0$$

(5) $$q_{i}^{-}-q_{i}^{0}\leq 0$$

(6) $$Q_{j}^{+}-Q_{j}^{0}\geq 0$$

(7) $$Q_{j}^{-}-Q_{j}^{0}\leq 0$$

(8) $$w_{i}^{+}=2-\frac{q_{ij}^{obs}-\min_{i\in S_{j}}\left(q_{ij}^{obs}\right)}{\max_{i\in S_{j}}\left(q_{ij}^{obs}\right)-\min_{i\in S_{j}}\left(q_{ij}^{obs}\right)}$$

(9) $$w_{i}^{-}=1+\frac{q_{ij}^{obs}-\min_{i\in S_{j}}\left(q_{ij}^{obs}\right)}{\max_{i\in S_{j}}\left(q_{ij}^{obs}\right)-\min_{i\in S_{j}}\left(q_{ij}^{obs}\right)}$$

(10) $$Q_{j}=\sum_{i\in S_{j}}q_{ij}$$

where $q_{i}$ and $q_{i}^{0}$ are the optimized and initial quantities (in grams) of food category *i* (e.g., beef), respectively, $Q_{i}$ and $Q_{i}^{0}$ are the optimized and initial quantities (in grams) of food group *j* (e.g., meat), respectively, and each of the *n* = 79 food categories belongs to one of the *m* =13 food groups. The technical variables $q_{i}^{+}$ and $q_{i}^{-}$ are introduced to distinguish between increases and decreases in consumption of food category *i*, respectively: $q_{i}=q_{i}^{+}$ if $q_{i}>q_{i}^{0}$ and 0 otherwise; $q_{i}=q_{i}^{-}$ if $q_{i}<\;q_{i}^{0}$ and 0 otherwise. The same applies to $Q_{j}^{+}$ and $Q_{j}^{-}$ at the food group level. It is necessary to introduce those somewhat cumbersome notations to impose different penalties for increases or decreases of more or less popular foods.

This original formulation of the objective function combines several attractive features that have been outlined in previous studies:

• First, the objective function minimizes both categorical and group deviations from the current diet, based on the approach of Barré et al. [25]. Hence, mycoprotein is not only added as a separate category but also as part of the meat group in Equation *1.* This is important, as intracategory substitutions among closely related products with similar functional properties (e.g., poultry or mycoprotein meat alternatives for beef) are likely to be easier than intercategory substitutions (e.g., porridge for beef).
• Second, the choice of a quadratic form imposes an increasing penalty on larger quantity changes for any food. This assumes that small changes in consumption of many foods are relatively more acceptable than large changes in the consumption of a few categories at the population level [20].
• Third, the change in intake of each food category/group is standardized by dividing it by the standard deviation (SD) of its initial intake ($\sigma_{i}$ and $\sigma_{j}$) in the dietary survey [16,26]. This converts all foods to the same unit of measurement, thus making the magnitude of changes comparable across food categories or groups. As opposed to dividing by the baseline amount, which would also standardize the food categories to the same unit, division by the SD expresses the change relative to the typical variation in the population. This implies that foods with relatively larger natural variation in the population can change while being penalized relatively less than other foods. For mycoprotein, the SD is estimated from the average SD of 5 common meat categories (beef, pork, poultry, sausages, and meat cuts).
• Finally, penalty weights ($w_{i}$ and $z_{j}$) are added to each food category and food group, which determine how their changes are differentially penalized during the optimization. Following the approach of Van Dooren et al. [21], the value of $w_{i}$ for each category is determined on the basis of the normalized value of the food’s initial intake quantity, which serves as a proxy for the food’s popularity among consumers (Equations *8* and *9*). The normalization is done among categories belonging to the same food group, where in Equation *10*, $q_{ij}$ means that food category *i* belongs to food group *j*, which is also part of the subset $S_{j}$. This ensures that only food categories that belong to the same group and tend to be consumed in quantities of a similar order of magnitude are compared with each other. The weight $w_{i}$ is directionally dependent (denoted as $w_{i}^{+}\;and\;w_{i}^{-}$) to reflect an assumption that people prefer to increase the consumption of popular foods rather than that of unpopular foods, whereas the consumption of unpopular foods is easier to decrease than that of popular foods. All values range from 1 to 2, where a higher value means that such a food change is less preferred (i.e., more penalized). Because the initial intake of mycoprotein is assumed to be 0 in this study (as it does not initially appear in the dietary survey), it is treated as the least popular food in the meat group, with $w^{+}=2$ and $w^{-}=1$. The values of the weights $z_{j}$ are set to 2 for all food groups, reflecting an assumption that substitutions between groups are more difficult than within groups.

We solved this optimization model using the “quadprog” package in R [27].

#### The constraints

A set of nutritional constraints defines the recommended or safe daily nutrient intake to ensure the nutritional adequacy of all optimized diets. We sourced the list of constraints from Irz et al. [28], who also studied the adult Finnish population. The list includes 30 constraints for macronutrients, 13 for vitamins, and 18 for minerals, defined on the basis of recommended or safe daily intakes drawn from the Nordic Nutrition Recommendations 2012, Finnish Nutrition Recommendations 2014, and WHO’s protein and amino acid recommendations, for males and females aged 18–64 y separately (see Supplemental Table 1 and Irz et al. [28] for more details). The constraint for energy is set constant at its observed dietary intake level. To keep the optimized diet within the observed intake range for the population of interest, we added feasible consumption constraints that limit the optimal intake of each food category within the 5th to 95th percentile of its observed consumption, hence adopting the assumption of Mazac et al. [8]. The constraint for mycoprotein was set at the food group level, where the consumption of mycoprotein and other meat categories was below the 95th percentile of the initial meat group consumption, as similarly assumed by Salomé et al. [16]. The “water” category was fixed at its observed intake value [20], and the mean observed intake replaced the zero initial value at the 95th percentile for some categories (e.g., game and lamb). In addition, we constrained the total food mass within ±20% of its initial level [29]. Finally, the GHGE constraint sets the maximum GHGE level of the optimized diet to form a set of GHGE-reduced dietary scenarios (see section “Optimization strategy”).

#### Optimization strategy

Starting from the current average Finnish diet (“Obs” scenario), a nutritionally adequate diet (“NUTR” diet) was first optimized by adding only the nutritional and feasible consumption constraints. The scenario described the potential of mycoprotein for improving the nutritional profile of the Finnish diet. The GHGEs of the “NUTR” diet were then reduced in a stepwise manner (“NUTR + GHGE-X%” scenarios, where “GHGE-X%” denotes the X% reduction in dietary GHGEs relative to the “NUTR” scenario), by imposing increasingly stringent GHGE constraints while keeping the total consumption of the meat group (i.e., meat plus mycoprotein) constant. This allowed for an assessment of the role of mycoprotein in maintaining meat group consumption levels while achieving lower GHGE impact diets. We ran the optimizations separately for an average male and female by using sex-specific nutritional constraints and dietary intakes.

#### Sensitivity analysis

We performed several sensitivity analyses to investigate the robustness of the simulated substitutions within the meat group to changes in key assumptions of the model. First, we ran the model with the nutritional profile of market-available mycoprotein-based meat alternatives (“mycoprotein product”). This gives a more realistic but also less desirable nutritional composition as compared with mycoprotein as an ingredient used in the baseline model because additional ingredients (e.g., sodium and egg albumin) are combined to improve the taste of the product [30]. Second, we removed all penalty weights on food categories to assess their effectiveness in bringing more realism and nuance in food preferences. Then, the sensitivity analysis considered how altering the GHGE coefficient of mycoprotein by ±50% would affect its inclusion under each “NUTR+GHGE” scenario. Although we assumed the baseline value represented the average GHGE coefficient of mycoprotein, in practice, this value is uncertain depending on energy use and processing methods [30]. Finally, to understand how results would change using different functional forms of the objective function, we ran the same simulations for the relative-piecewise-linear form of the objective function (linear models, where deviation is measured by the absolute value $\left|\frac{q^{i}-q_{0}^{i}}{q_{0}^{i}}\right|$). The assumption of this model is that larger changes in a small number of foods are considered more acceptable to the population of interest [19]. Supplemental Material: Mathematical Appendix details the model’s formulation, which was solved using the lpSolve package in R [31].

### Data

Following the method of Gazan et al. [32], we compiled a database with 79 food categories (in 13 food groups), covering different sustainability metrics of foods (see Supplemental Data). The number of categories was based on the GHGE dataset, which contains the smallest number of foods available across datasets.

#### Dietary intake and food nutrient composition data

The Finnish average daily dietary intake data were collected from the FoodEx2 database of the European Food Safety Association (EFSA), based on the FinDiet2017 survey [33]. We selected food items at the EFSA FoodEx2 L6 exposure hierarchy that contains 613 food items for males and 643 for females aged 18–64 y and then aggregated them into 78 food categories. We also multiplied the consumption data of each food item by a yield factor from the literature [8,34] to ensure that they are expressed in “as consumed” form (e.g., meat from raw to cooked mass and legumes from dried to cooked, wet mass). We added mycoprotein as a separate food category in the meat group with an initial consumption value set to 0 g. We matched each food item in EFSA with a set of nutrient composition coefficients, where the macro- and micronutrients were collected from the National Food Composition Dataset Fineli [35], and essential amino acids from the USDA’s FoodData Central Database [36]. The nutrient composition of each food category was calculated as the weighted average of the nutrient composition of food items within that category, with weights based on the current intake of each food item. For mycoprotein, the nutrient composition coefficients used in the main analysis were sourced from Mazac et al. [8], and the nutritional data on mycoprotein products used in the sensitivity analysis were calculated from the mean nutrient content of mycoprotein-based meat alternative products found in 3 nutrient composition datasets: Fineli (Finland), Livsmedelsverket (Sweden), and Matvaretabellen (Norway).

#### GHGE data

We obtained the GHGE coefficients for each food category from Mazac et al. [37], who calculated the global warming potential (GWP) of 81 Finnish foods at the ingredient level using life cycle assessment, with a functional unit of “1 kg of ready-to-eat food” and a system boundary of “cradle to plate.” Some adjustments were made for this study, for example:

• For cereal-based foods (e.g., bread), the GHGE coefficient was calculated from the weighted sum of its ingredients’ emission based on the recipe from Fineli [35].
• Raw meat and dried cereals were multiplied by a yield factor to derive the GHGE coefficient of cooked foods from literature [8,34].

We sourced the GHGE coefficient data of mycoprotein from Mazac et al. [38], whose estimation approach was consistent with that used by Mazac et al. [37] for other food categories. We adjusted the GHGE coefficient data, originally based on European consumers, to the Finnish case by modifying the electricity source, which is an important factor influencing the environmental impact of mycoprotein [12,30]. We assumed that the electricity use accounted for 60% of the GWP of mycoprotein (based on the estimation of Finnigan et al. [39]) and collected the GWP of electricity mix from the Ecoinvent 3 Life Cycle Inventory database.

#### Food price data

We utilized data on 259 food and drinks categories from Statistics Finland’s Household Budgetary Survey (HBS) to calculate food prices. Specifically, we calculated the price of 259 HBS categories by dividing average expenditure by quantity purchased and then matched with 78 food categories of this study whenever possible. For categories that are not covered by the HBS (e.g., alcoholic drinks and plant-based drinks), price data were collected online from 2 main supermarkets in Finland (K market and Prisma), and an inflation-adjusted average price was calculated. For mycoprotein, we calculated the average price from mycoprotein products sold under the Quorn brand.

## Results

### Substitutions within the meat group

**FIGURE 1:**
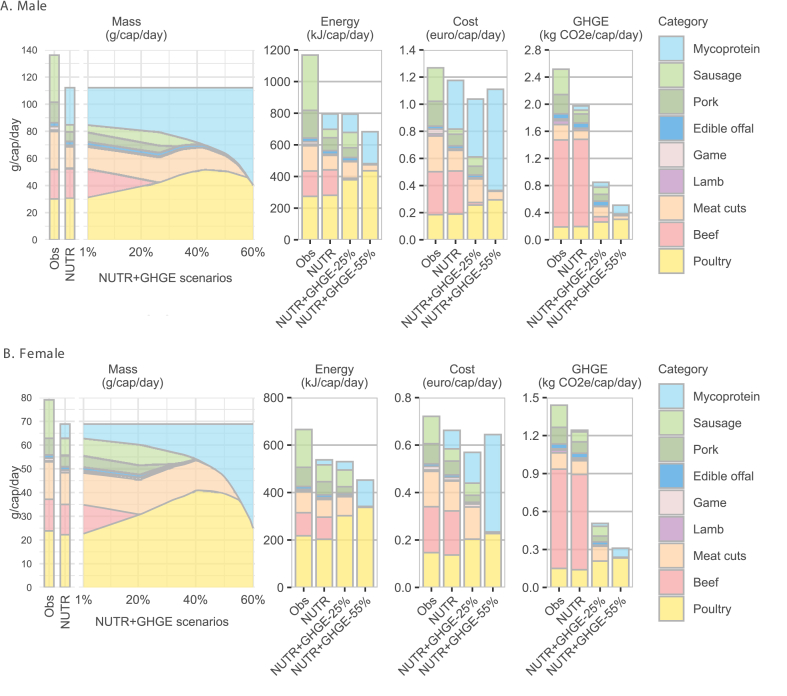
Categorical mass change within the meat group, and their contribution to energy, cost, and greenhouse gas emissions (GHGEs) of the meat group for the observed diet and different optimized diets, for (A) males and (B) females. The “Meat cuts” category refers to marinated and cured meat items (e.g., ham and bacon). "Obs" refers to the current average Finnish diet; NUTR, denotes the nutritionally adequate diet; The NUTR+GHGE, scenarios are obtained by reducing the GHGEs of the nutritionally adequate diet in a stepwise manner; "cap/day" denotes per capita per day; CO2e is the abbreviation of CO2 equivalent.

We first examined substitutions within the meat group across the different scenarios and their implications for diet cost and GHGEs, as shown in Figure 1. In the “NUTR” diet scenario, overall consumption of the meat group decreased by 18% for an average male and by 13% for an average female. This reduction was driven primarily by a significant decline in processed meats (sausages and meat cuts), partially offset by an increase in mycoprotein (27.3 g for males and 5.9 g for females). The level of red and processed meat reached 51 g for males and 39.2 g for females, which is close to the maximum levels recommended in the new Finnish dietary guideline [23], making the optimized meat group potentially healthy from an epidemiological perspective. As progressively stricter GHGE reductions were imposed on the “NUTR” diet, consumption of beef, pork, sausage, and meat cuts declined further, and these foods were gradually replaced by poultry and mycoprotein. Notably, consumption of poultry increased faster than that of mycoprotein up to the 40% GHGE reduction scenario, after which the trend reversed, with mycoprotein consumption increasing relatively more. Expressed in terms of energy, the changes within the meat group are similar to the changes in terms of mass except for mycoprotein and poultry. This is explained by the fact that mycoprotein has a lower energy content than poultry (355.6 kJ/100 g compared with 911.7 kJ/100 g). Compared with poultry, mycoprotein offers advantages in terms of GHGEs but is more costly to incorporate into the diet. For example, in the “NUTR + GHGE-55%” scenario for an average male, mycoprotein accounts for a significantly larger share of the total cost of the meat group than that of poultry (67% compared with 27%), whereas it contributes a smaller share of the group’s GHGEs (25% compared with 59%). The reduction in the quantity of beef consumed is the main cause of the reduction in cost and GHGEs of the meat group, which is explained by its high emission intensity (5.9 kg carbon dioxide equivalents/100 g) and relatively high price (1.5 €/100 g).

### Substitutions between food groups

**FIGURE 2:**
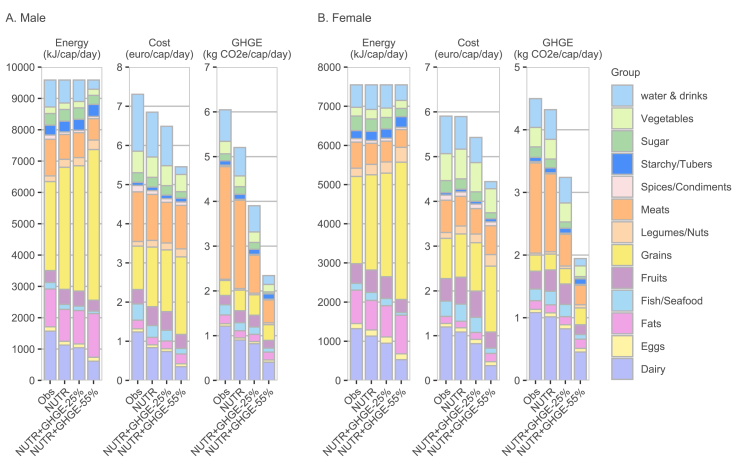
Food group contribution to energy, cost, and greenhouse gas emissions (GHGEs) of the observed "Obs" diet, nutritionally adequate “NUTR” diet, and “NUTR” diet with 25% and 55% GHGE reduction, for (A) males and (B) females. NUTR, nutritionally adequate diet; cap/day, per caipta per day; CO2e, CO2 equivalent.

**FIGURE 3:**
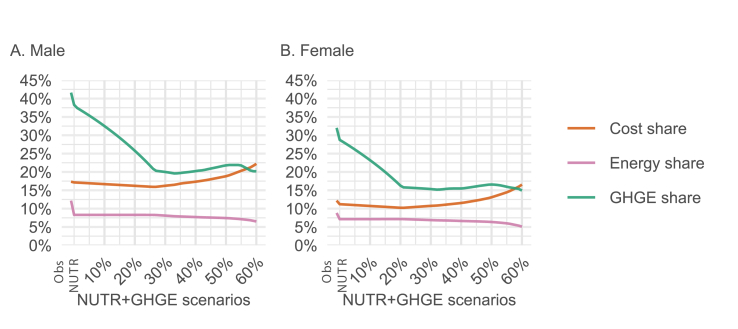
Meat group contribution to energy, greenhouse gas emissions (GHGEs), and costs of the optimized diets for (A) males and (B) females. “NUTR+GHGE” refers to scenarios where the GHGE of the nutritionally adequate diet is reduced in a stepwise manner. NUTR, nutritionally adequate diet; Obs, the current average Finnish diet.

We then investigated the energy composition of the optimized diets as well as their properties in terms of cost and GHGEs for all food groups (Figure 2) and specifically for the meat group (Figure 3). These graphical results are complemented by detailed within-group changes in the physical quantity of each food category in Supplemental Tables 2 and 3.

Figure 3 shows that the energy share of the meat group first declines in the “NUTR” scenario and then remains fairly constant as more stringent GHGE reductions are imposed and mycoprotein consumption increases. Meanwhile, the meat group’s contribution to dietary GHGEs initially drops sharply, before leveling off at ∼20% of total GHGEs for males and 15% for females. In terms of dietary cost, the meat group’s share initially decreases slightly, followed by a gradual increase for both sexes.

For other food groups (Figure 2), energy intake from the dairy group declines markedly, from 16.4% (17.5%) in the current diet to 6.4% (7.0%) in the GHGE-55% scenario for males (females), primarily because of the elimination of cheese and fermented milk categories. This reduction leads to a corresponding decrease in GHGEs from the dairy group, even though the share of that food group in total dietary GHGEs remains considerable [17.4% (23.2%) for males (females) in the GHGE-55% scenario]. The water and drinks, fruit, and fish groups also decrease in importance in terms of energy, with parallel declines in GHGEs and cost contributions. The energy loss from these food groups is largely offset by a substantial increase in grain consumption [e.g., rise of 69.2% (male) and 57.4% (female) from the current diet to the GHGE-55% diet]. This includes increased intake of breakfast cereals, rye bread/rolls, and multigrain bread/rolls. Consumption of legumes and starchy foods also increases slightly in terms of energy; however, their contributions to dietary GHGEs and cost remain minimal in all simulated diets.

### Food group contributions to nutrient intakes in the optimized diets

To further examine the nutritional characteristics of the simulated dietary changes, Supplemental Figures 1 and 2 present how each food group contributes to the intake of binding nutrients and nutrients for which the meat group contributes >20% of intake in the observed or optimized diets. For a male, in the current diet, meat is an important source of vitamin A (26%), thiamin/B1 (27%), niacin/B3 (29%), vitamin B6 (28%), vitamin B12 (32%), iron (20%), zinc (28%), and selenium (26%). In the optimized diets, the inclusion of additional mycoprotein is sufficient to maintain the meat group’s contribution to zinc and selenium intake. However, for intakes of vitamin A and iron, the reduction in meat group consumption is offset by intergroup substitutions, with additional consumption of fats and vegetables (for vitamin A) and grains (for iron). In addition, intakes of vitamins B1, B3, B6, and B12 from the meat group decrease significantly. Although meat is not initially the main source of riboflavin/B2 (15%) and dietary fiber (0%), those shares increase to 32% for riboflavin and 9% for dietary fiber in the “NUTR + GHGE-55%” diets that contain considerable amounts of mycoprotein. For sodium, a nutrient whose intake exceeds the upper recommended level, the contribution from the meat group decreases from 20% in the observed diet to only 4% in the “NUTR + GHGE-55%” diet.

The nutritional composition of the meat group in the optimized diets reveals an improved fatty acid profile, characterized by higher levels of essential fatty acids combined with less total fat and saturated fatty acids (SFAs). The total dietary protein intake is well above the recommended minimum level in all scenarios, although the contribution of the meat group to total protein intake declines by 6% from the current diet to the “NUTR + GHGE-55%” diet.

For females, the relative contributions of the food groups to nutrient intakes are broadly similar to those described for males, but the magnitude of the changes is, in general, more modest.

### Sensitivity analysis

**FIGURE 4:**
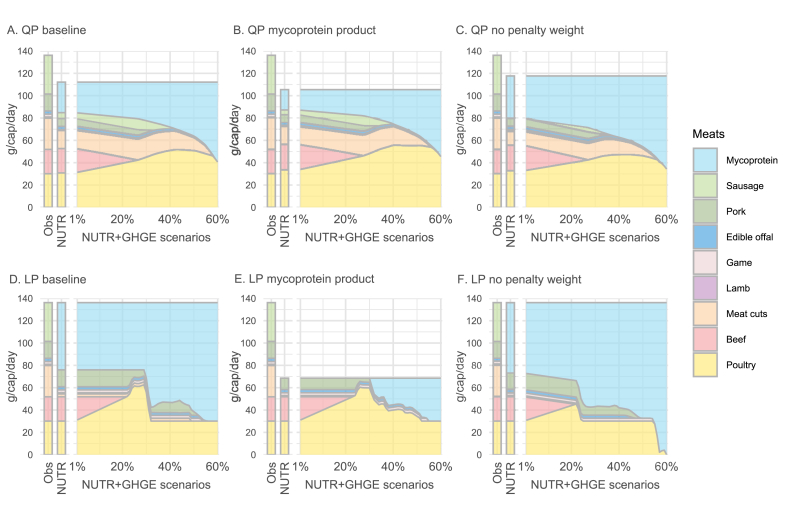
Sensitivity analysis results for the male population displaying the categorical changes within the meat group for (A, D) baseline model settings; (B, E) a modified nutritional profile of mycoprotein; (C, F) removal of penalty weights on each food category, for quadratic models (A–C) and linear models (D–F). “NUTR+GHGE” refers to scenarios where the GHGE of the nutritionally adequate diet is reduced in a stepwise manner. NUTR, nutritionally adequate diet; Obs, the current average Finnish diet; QP, quadratic programming model; LP, piecewise linar programming model.

**FIGURE 5:**
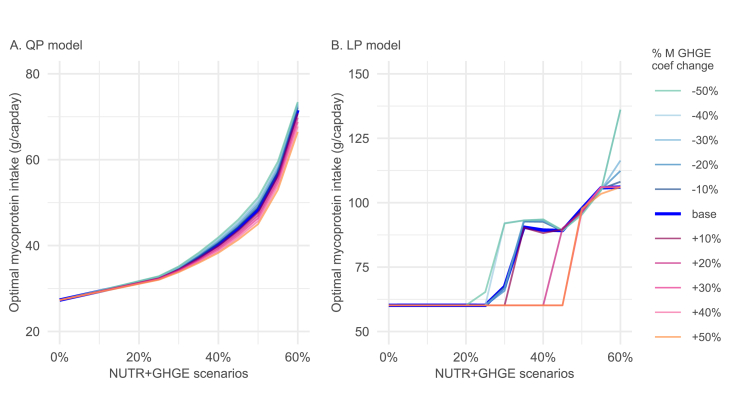
Sensitivity analysis results (for male population): the inclusion of mycoprotein in simulated diets, considering the baseline greenhouse gas emission (GHGE) coefficient of mycoprotein, and ±50% changes in the value of the coefficient, for (A) quadratic model and (B) linear model. “NUTR+GHGE” refers to scenarios where the GHGE of the nutritionally adequate diet is reduced in a stepwise manner. QP, quadratic programming model; LP, piecewise linar programming model.

FIGURE 4, FIGURE 5 show how the inclusion of mycoprotein and categorical shifts within the meat group vary under alternative assumptions in the baseline model, for an average male. First, we adjusted the model to use nutritional coefficients based on the nutritional composition of existing mycoprotein products (Figure 4B), rather than mycoprotein as an ingredient food as used in our baseline model (Figure 4A). The revised nutritional coefficients result in a lower inclusion of mycoprotein in the “NUTR” diet than in the baseline model (18.4 g compared with 27.5 g), even though in the “NUTR+GHGE-60%” scenario, mycoprotein accounts for a similar share of the meat group (57% in the revised model compared with 63% in the baseline model). The similarity in substitution patterns across other meat categories between the 2 models indicates the robustness of the results.

Second, we investigated the influence of penalty weights on results by simulating diets in the absence of those weights, as shown in Figures 4A and 4C. In the “NUTR” scenario, mycoprotein accounts for a larger share of the meat group with the revised (no weights) model compared with the baseline model (32% compared with 24%). In the GHGE-reduced scenarios, the removal of the penalty weights results in larger increases in consumption of mycoprotein, reflecting its nutritional and environmental advantages over other meat options. This demonstrates that, as intended, penalty weights introduce a “reluctance” to consume new foods, significantly impacting substitutions across food categories.

We then assessed how a ±50% change in the GHGE coefficient of mycoprotein would affect its inclusion in the GHGE-reduced diets (Figure 5A), motivated by the large uncertainty surrounding the value of this parameter. The optimal mycoprotein intake obtained from the model with the altered GHGE coefficient differs only slightly from the corresponding intake in the baseline model, most notably in the scenarios with GHGE reductions under 35%. This suggests that the role of mycoprotein in achieving stringent dietary GHGE reductions is not highly sensitive to variations within this range of GHGE parameter.

Finally, the results of the quadratic model depicted in FIGURE 4, FIGURE 5A were compared with those derived from the piecewise linear model depicted in FIGURE 4, FIGURE 5B. In the baseline models (Figures 4A and D) and the “NUTR” scenario, the linear model incorporates more mycoprotein than the quadratic model, with mycoprotein accounting for 44% compared with 24% of the consumption of the meat group. As stricter GHGE reductions are gradually imposed, the linear model maintains a near-constant intake of mycoprotein until a threshold level of 30% GHGE reduction is reached, at which point intake increases sharply. The intake of poultry increases linearly before exhibiting abrupt fluctuations in a pattern that appears unrealistic. The linear models with altered assumptions (i.e., “mycoprotein product” and “no penalty weight”) also generate similar “corner solutions,” where the composition of the optimized meat group appears overly simplistic, lacking variety, and less acceptable compared with the more balanced diets simulated with the quadratic models. Despite these differences, quadratic and linear models also yield some consistent findings. For instance, both reveal that greater dietary GHGE reductions are associated with increased inclusion of mycoprotein in optimized diets (Figure 4), and that mycoprotein intake is more responsive to a decrease in the GHGE coefficient of mycoprotein than to an equivalent increase (Figure 5). Altogether, the substitution pattern favoring increased mycoprotein intake in nutritionally adequate, low-climate impact diets appears relatively robust to changes in the mathematical form of the objective function.

## Discussion

The simulated diets in this study indicate that mycoprotein could potentially support dietary transitions by functioning as a meat alternative. In an optimized nutritionally adequate diet, incorporating small amounts of mycoprotein requires only an 18% reduction in consumption of the meat group for a male, which is much less than what has been found to be needed in the absence of meat alternatives, such as a 35.4% reduction in the study by Irz et al. [28]. The results for an average female are qualitatively similar but quantitatively less pronounced. Achieving higher GHGE reduction targets while maintaining meat group consumption to its level in the nutritionally adequate NUTR diets requires increased consumption of mycoprotein, which substitutes for meats due to its relatively lower GHGE coefficient, thereby contributing to an overall decrease in GHGEs from the meat group.

From a whole dietary perspective, where changes in other food groups are concerned, sustaining a high level of meat group consumption (i.e., close to current intake) with the introduction of mycoprotein as a meat alternate appears more advantageous for achieving moderate dietary GHGE reductions [e.g., the “NUTR+GHGE25%” diet that incorporates 32 g (10 g) of mycoprotein for an average male (female)]. This is because, when imposing moderate dietary GHGE reduction targets, the drastic decrease in the meat group’s share of dietary GHGEs (mainly from substituting beef with poultry and mycoprotein) reduces the adjustments required from other food groups. However, this effect plateaus as larger reductions in GHGEs are pursued. From a nutritional perspective, mycoprotein lacks certain micronutrients compared with meat (e.g., vitamins A, B1, B3, B6, and B12 and iron). Consequently, in diets with high GHGE reductions where mycoprotein accounts for a high share of the meat group, the overall contribution of the meat group to the total dietary intakes of these nutrients diminishes. This necessitates compensatory changes in other food groups to meet the constraints for binding nutrients (e.g., more vegetables and grains for vitamins A and B1, respectively). However, this issue is less pronounced when mycoprotein only accounts for a small share of consumption of the meat group in diets with moderate GHGE reductions.

Regarding the affordability of the diets, the cost of the meat group is 2.8%–23.0% less than the cost of the meat group in the current diet, despite the fact that the price of mycoprotein is similar to that of beef (13.1 €/kg compared with 14.6 €/kg, respectively), which is also twice the average price of poultry. The cost reduction arises from the partial substitution of expensive red and processed meats with both similarly priced mycoprotein and more affordable chicken. Thus, the overall diet cost might not represent a major barrier to the adoption of lower-impact diets that include mycoprotein. Nevertheless, the actual behavioral change remains a challenge, as the high price of mycoprotein may deter some consumers in the first place [40]. At that level, it is encouraging that the main drivers of production costs of mycoprotein and related technological barriers have been identified and are actively being addressed; for example, cocultivation (mixed fermentation) methods show promising potential for enhancing the productivity and quality of single-cell protein [41]. Thus, there is a strong possibility that mycoprotein might become more cost-competitive and attractive for consumers in the future.

From a methodological perspective, we have extended current approaches for optimizing culturally acceptable diets that incorporate new foods. Our model captures cultural acceptability not only through the objective function, which minimizes deviation from the current diet, but also through penalty weights that reflect consumers’ stronger preferences for meat over meat alternatives, which are only imperfect substitutes [18]. The penalty weight approach was shown to be effective in capturing the difficulty of replacing meat with alternatives. Hence, the sensitivity results revealed that, in the absence of penalty weights, mycoprotein’s inclusion in a nutritionally adequate diet would be 40% more than that simulated with penalty weights. This effect was also evident in the finding that poultry (a relatively more popular food) was utilized more extensively than mycoprotein to achieve stringent GHGE reduction targets in most scenarios, despite the fact that mycoprotein is 64% less emission-intensive than poultry. Unlike previous studies, such as those by by Mazac et al. [8] and Salomé et al. [16], that quantified the maximum potential benefits of incorporating new foods into diets, our results offer more realistic insights into their roles in the transition toward sustainable diets. This represents valuable evidence for decision makers to weigh the importance of new foods in future dietary recommendations because consumer acceptance is an important obstacle to the adoption of those foods [11] and therefore a key element determining the extent to which the potential benefits of new foods can be realized.

In addition, our study offers new methodological insights, particularly regarding the practical implications of choosing between quadratic and linear objective functions in diet optimization. The sensitivity analysis supports previous findings in the literature that quadratic models favor small changes across a wide range of foods, whereas linear models often result in more drastic changes concentrated in a smaller set of items [19,42]. This pattern also applies to new foods: the inclusion of mycoprotein in the simulated diets is relatively less pronounced with the quadratic model compared with its linear equivalent. Furthermore, the inclusion of mycoprotein in the diet is very sensitive to parameter changes in linear models, which is an undesirable feature to capture the role of new foods in diets, given the fact that the environmental and nutritional properties of those new foods are often uncertain. On the basis of these findings, we conclude that quadratic models are preferable to linear models for optimizing diets that include new foods, as they produce more realistic substitution patterns and are less sensitive to parameter uncertainty.

Nevertheless, we must acknowledge several limitations of this study that may guide future research. One limitation is that the nutritional profile of mycoprotein used in the main analysis was based on the corresponding ingredient itself, rather than the mycoprotein-based products available to consumers in the Finnish market. This issue was studied in the sensitivity analysis, but it must be acknowledged that the available nutritional data were limited, with large variations depending on product type and the ingredients combined with mycoprotein in the final product [30]. The simulated diets tended to incorporate less mycoprotein product than the pure mycoprotein ingredient, suggesting that our main results may somewhat overestimate the nutritional benefits of including mycoprotein in diets. However, this potential overestimation also highlights opportunities for improvement, which should encourage manufacturers to enhance the nutritional profile of mycoprotein products. Thus, more detailed nutritional data on commercially available mycoprotein products are needed to enable more accurate assessments, particularly in identifying which specific meat categories these products would compete with and replace.

The treatment of cultural acceptability in our model, especially the use of penalty weights that are based on observed food intakes, also represents a necessary simplification and may not fully capture differences in willingness to substitute meats across sexes or age groups [43]. In addition, whether our approach of fixing meat group consumption at its level in the “NUTR” diet has a strong influence on the optimized diets needs careful inspection in the future. More sophisticated methods to capture consumer preferences exist—for example, in economics, stated preference methods [40] derive consumers’ willingness to pay or intention to substitute meat from survey data, whereas revealed preference methods [44] infer consumer preferences from actual purchase data. In terms of nutrition, these methods provide distinct yet complementary insights. Economic valuation helps assess how nutritional information influences consumer decisions, which can inform diet optimization approaches that propose diets fulfilling a broad set of nutritional constraints and other dietary targets, such as GHGE reductions [3]. Thus, our model should be seen as a foundational starting point. Future research could improve the treatment of dietary acceptability by, for example, incorporating economic valuations (e.g., constructing penalty weights based on people’s measured preferences), thereby capturing more realistic consumer food choices without compromising the rigor of the nutritional analysis.

Although our optimization focuses solely on nutritional adequacy, adding food-based health constraints could substantially alter the simulated diets. For instance, the intake of fruits and vegetables in the “NUTR” and “NUTR+GHGE-55%” diets for an average male is 346.2 g and 298.6 g/d, respectively, which falls well below the >500 g/d recommended in the Finnish food-based dietary guidelines [23]. Imposing such a constraint would require significant adjustments, particularly under stringent GHGE targets, as the relatively high GHGE intensity of fruits and vegetables in Finland—driven partly by imports—would shift the mitigation burden to other food groups. By contrast, the consumption of red and processed meat in the “NUTR” diet is already close to the recommended limit of 50 g/d, suggesting that an additional constraint on this group would bind only marginally, if at all.

Finally, our study does not address other important concerns that influence the role of meat substitutes in sustainable diets, underscoring the need for future research. One such concern is that meat alternatives might not entirely replace meat as intended but simply be added to current consumption of meat, thereby rendering their aggregated environmental impacts uncertain. As producers of meat alternatives seek to lower prices and expand into new markets to increase sales, there is a risk that people will increase their consumption of these alternatives without reducing meat consumption by much. This would lead to overconsumption, which could offset the environmental benefits of alternative protein consumption—a result broadly consistent with the Jevons paradox [45]. This concern is partially supported by Neuhofer and Lusk [46], who found, using United States household scanner data, that increased purchases of plant-based meat alternatives did not lead to a reduction in ground meat purchases. More empirical evidence is needed to clarify this relationship and, hence, better inform consumers while also addressing risk of excess protein consumption. In addition, our study did not capture the substitution between mycoprotein and traditional plant-based meat alternatives (e.g. tofu), which might also yield important implications for protein balance that needs further inspections. The other limitation is that the assessed dietary costs are ex post and static in the sense that prices are assumed to be constant at their current levels. Thus, we abstract from the potentially substantial but unknown adjustments in prices that may occur in the future as a result of dietary changes. A large uptake of mycoprotein at the population level would boost demand for mycoprotein, but whether this shift in demand would raise the price is an open question that depends on the elasticity of supply of mycoprotein (see Capacci and Mazzocchi [47] for the argument as applied to the consumption of fruits and vegetables). Estimation of this type of price effects lies beyond the scope of our study. It is also important to recognize that consumer-driven diets represent only one component of the broader food system, which evolves as the result of close interaction with other supply-side actors, such as traditional meat producers, retailers, processors, and regulators. These interactions influence both the types and quantities of meat alternatives that could be produced and the prices at which they are offered, all questions that are closely tied to consumer behavior. Therefore, to understand the food system implications of adopting meat alternatives, more research is needed to explore the interconnections between dietary change, production, and trade. One promising avenue would be to link dietary optimization models with general equilibrium [48] or partial equilibrium [7] models.

To conclude, this study proposed a novel optimization model that incorporates meat alternatives to characterize nutritionally adequate and GHGE-reduced diets and gives due consideration to consumer acceptability. The practical use of the approach was illustrated by applying the model to Finnish data, which allowed exploration of the potential role that mycoprotein could play in the transition toward nutritionally adequate, affordable, and climate-friendly diets. The model can be readily adapted to generate realistic dietary scenarios that include various emerging meat alternatives and other novel foods, thereby enhancing our understanding of their potential in the pursuit of sustainable diets.

## Author contributions

The authors’ responsibilities were as follows – YF, XI: designed the research; YF: compiled the data, coded the models, ran the simulations, produced the figures, and wrote the first draft; XI: provided codes of diet optimization models that were adapted by YF, data, and feedback on all versions of the paper and edited the manuscript; and both authors read and approved the final manuscript.

## Data availability

Data described in the manuscript are available in the Supplemental materials: Supplemental Data. R codes for the baseline quadratic and piecewise linear model are freely available without restrictions in Mendeley Data [49] at https://doi.org/10.17632/b9nm452g7h.1.

## Declaration of generative AI and AI-assisted technologies in the writing process

During the preparation of this work, the authors used DeepL to proofread the language. After using this tool, the authors reviewed and edited the content as needed and take full responsibility for the content of the publication.

## Funding

Open access was funded by Helsinki University Library.

## Conflict of interest

The authors report no conflicts of interest.
