# Supplementary material for: Designing Culturally Acceptable, Nutritious, and Low Environmental Impact Finnish Diets with Mycoprotein: A Novel Optimization Approach
Source: Curr Dev Nutr. 2025 Sep 22;9(11):107559. doi: 10.1016/j.cdnut.2025.107559 (PMC12615728; doi:10.1016/j.cdnut.2025.107559)
Supplement: multimedia component 1 [file mmc1.pdf]

Supplemental Materials for manuscript: Designing culturally acceptable, nutritious and low environmental impact Finnish diets with mycoprotein: a novel optimization approach

Authors: Yue Fu, Xavier Irz*

Table of contents

Supplemental Material: Mathematical Appendix

Formulation of the piecewise linear model 2

Supplemental Tables

Supplemental Table 1: Nutrient recommendations applied in optimization model, and the nutrient intake of the current diet (Obs), in per person per day 4

Supplemental Table 2: Food categorical intake (g/cap/day) of the observed diet and three optimized diets (“NUTR”, “NUTR+GHGE-25%” and “NUTR+GHGE-55%”) for an average Finnish female 6

Supplemental Table 3: Food categorical intake (g/cap/day) of the observed diet and three optimized diets (“NUTR”, “NUTR+GHGE-25%” and “NUTR+GHGE-55%”) for an average Finnish male 9

Supplemental Figures

Supplemental Figure 1: Nutritional contributions of food groups to total nutrient intakes, for selected nutrients (Female) 12

Supplemental Figure 2: Nutritional contributions of food groups to total nutrient intakes, for selected nutrients (Male) 13

Supplemental Data

Supplemental Data 1: data for different sustainability etrics of foods by category (female) 14

Supplemental Data 2: data for different sustainability etrics of foods by category (male) 20

Supplemental Material: Mathematical Appendix

Formulation of the piecewise linear model

The objective function for a piecewise linear model takes the following form:

$$Min \sum_{i=1}^{n} w_i \times \left|\frac{q_i - q_i^0}{\sigma_i}\right| + \sum_{j=1}^{m} z_j \times \left|\frac{Q_j - Q_j^0}{\sigma_j}\right| \qquad (S1)$$

where q_i and q_i^0 are the optimized and initial quantity of food category i (e.g., beef), Q_j and Q_j^0 are the optimized and initial quantity of food group j (e.g., meat), and each food category belongs to one of the m food groups.

w_i and z_j are the penalty weights imposed on each food category and food group. They are similar to those introduced in the quadratic model. In particular, the value of w_i is directionally dependent, and is calculated as:

$$w_i^+ = 2 - \frac{q_{ij}^{obs} - \min(q_{ij}^{obs})}{\max(q_{ij}^{obs}) - \min(q_{ij}^{obs})} \qquad (S2)$$

$$w_i^- = 1 + \frac{q_{ij}^{obs} - \min(q_{ij}^{obs})}{\max(q_{ij}^{obs}) - \min(q_{ij}^{obs})} \qquad (S3)$$

For the value of z_j, $z_j^+ = z_j^- = 2$ for all food groups.

As the objective function (equation (S1)) is piecewise linear rather linear, to apply the linear programming package “lpsolve” in R, the absolute values in equation (S1) need to be removed. To achieve this, additional constraints and two auxiliary variables (u_i and v_i) are added to transform the first and the second part of the objective function, respectively:

$$-u_i \le w_i\left(\frac{q_i - q_i^0}{\sigma_i}\right) \le u_i \qquad (S4)$$

$$-v_i \le z_j\left(\frac{Q_j - Q_j^0}{\sigma_j}\right) \le v_i \qquad (S5)$$

which can be further written as (with separated weights):

$$w_i^+ (\frac{q_i - q_i^0}{\sigma_i}) \le u_i \qquad (S6)$$

$$-u_i \le w_i^-\left(\frac{q_i - q_i^0}{\sigma_i}\right) \qquad (S7)$$

$$z_j^+(\frac{Q_j - Q_j^0}{\sigma_j}) \leq v_i \tag{S8}$$

$$-v_i \leq z_j^- \left(\frac{Q_j - Q_j^0}{\sigma_j}\right) \tag{S9}$$

Equations (S6) and (S8) take effect when the optimized intake is increased from the current intake ($q_i - q_i^0 \geq 0, Q_j - Q_j^0 \geq 0$), equations (7) and (9) are active when the optimized intake is decreased from the current intake ($q_i - q_i^0 \leq 0, Q_j - Q_j^0 \leq 0$). Through equations (S6-9), the constraints imposed on $\frac{q_i - q_i^0}{\sigma_i}$ and $\frac{Q_j - Q_j^0}{\sigma_j}$, together with their value changes, can be transferred to u_i and v_i , and the objective function is transformed to the following linear form:

$$Min\left(\sum_{i=1}^{n} u_i + \sum_{i=1}^{m} v_i\right), \; u_i \geq 0\,, v_i \geq 0 \tag{S10}$$

Thus, the decision variables of this optimization model in R are: q_i, Q_i, u_i, v_i.

Supplemental Tables

Supplemental Table 1: Nutrient recommendations applied in optimization model, and the nutrient intake of the current diet (Obs), in per person per day. The energy constraint is set constant at the level of the current diet. Values highlighted in red mean that the initial nutrient intake fails to meet the nutritional recommendations. Source: Irz et al. (2024) and authors' own calculations.

Nutrient	Unit	Male (18-64)			Female (18-64)		
		Min	Max	Obs	Min	Max	Obs
**Vitamins and Minerals**							
Vitamin A	µg	900		948.8	700	3000	725.2
Vitamin D	µg	10	100	11.8	10	100	9.0
Vitamin E	mg	10	300	11.8	8	300	10.2
Thiamin/B1	mg	1.4		1.5	1.1		1.2
Riboflavin/B2	mg	1.6		2.0	1.3		1.6
Niacin/B3	mg	19		39.0	15		29.4
Vitamin B6	mg	1.6		2.2	1.2		1.8
Folate	µg	300		282.1	300		242.6
Vitamin B12	µg	2		6.7	2		4.8
Vitamin C	mg	75		116.1	75		117.6
Calcium	mg	800	2500	1188.8	800	2500	993.0
Phosphorus	mg	600	3000	1696.2	600	3000	1345.2
Magnesium	mg	350		418.5	280		345.4
Potassium	g	3.5		4.0	3.1		3.4
Iron	mg	9	25	12.1	10.0	25	10.0
Zinc	mg	9	25	12.7	7	25	9.6
Iodine	µg	150	600	230.2	150	600	188.8
Selenium	µg	60	300	87.9	50	300	72.5
Copper	mg	0.9	5	1.4	0.9	5	1.2
Sodium	mg	575	2400	3405.7	575	2400	2635.7
**Macronutrients**							
**Fat & Fatty acids**							
Total fat	E%	25	40	38.1	25	40	37.2
Saturated fatty acids	E%		10	13.4		10	12.6
Cis-MUFA	E%	10	20	14.4	10	20	14.1
Cis-PUFA	E%	5	10	6.5	5	10	6.6

Nutrient	Unit	Male (18-64)			Female (18-64)		
		Min	Max	Obs	Min	Max	Obs
Alpha-linolenic acid	E%	1		1.3	1		1.3
Essential fatty acids	E%	3		6.1	3		6.1
Docosahexaenoic acid	mg	200		343.9	200		301.7
**Proteins**							
Proteins	E%	10	20	16.3	10	20	15.8
Proteins	g/kg bodyweight	0.83		1.1	0.83		1.0
Histidine	mg/kg bodyweight	12.4		33.5	12.4		30.0
Isoleucine	mg/kg bodyweight	24.8		53.1	24.8		48.9
Leucine	mg/kg bodyweight	48.4		93.4	48.4		85.9
Lysine	mg/kg bodyweight	37.2		78.3	37.2		70.8
Methionine + cysteine	mg/kg bodyweight	18.6		42.9	18.6		39.0
Phenylalanine + tyrosine	mg/kg bodyweight	31		97.3	31		90.2
Threonine	mg/kg bodyweight	18.6		46.6	18.6		42.7
Tryptophan	mg/kg bodyweight	5		14.2	5		13.1
Valine	mg/kg bodyweight	32.2		63.4	32.2		58.8
Total essential amino acids	mg/kg bodyweight	228		463.4	228		424.7
**Others**							
Carbohydrates (Total)	E%	45	60	40.4	45	60	41.8
Dietary fiber	g	35		21.5	25		19.2
Alcohol	E%		5	2.1		5	1.3
Energy	kJ			9586.4			7546.0

Reference:

Irz X, Tapanainen H, Saarinen M, Salminen J, Sares-Jäske L, Valsta LM. Reducing the carbon footprint of diets across socio-demographic groups in Finland: a mathematical optimisation study. Public Health Nutr. 2024;27(1):e98. Epub 2024/03/04. doi: 10.1017/S1368980024000508.7.5

Supplemental Table 2: Food categorical intake (g/cap/day) of the observed diet and three optimized diets ("NUTR", "NUTR+GHGE-25%" and "NUTR+GHGE-55%") for an average Finnish female. The cells in yellow/green means that the categorical intake reaches the minimum/maximum feasible consumption constraint.

Food category by group		Current	NUTR	NUTR+ GHGE-25%	NUTR+ GHGE-55%
Grains		286.3	310.5	327.7	424.7
	Rice and rice flour	41.7	36.1	22.7	0.0
	Wheat and wheat flour	8.4	8.1	9.0	12.1
	Rye and rye flour	0.1	0.1	0.1	0.1
	Other grain and grain flour	1.2	1.2	1.2	1.2
	Starch	1.0	1.0	1.0	1.0
	Wheat bread and rolls	13.9	14.4	16.2	22.9
	Rye bread and rolls	27.2	47.1	51.0	66.9
	Multigrain bread and rolls	33.2	50.7	55.6	75.5
	Pasta	20.5	19.0	19.4	8.5
	Noodles	8.3	0.7	2.4	5.3
	Breakfast cereal	77.6	88.9	96.5	151.0
	Fine bakerly wares	47.1	37.0	45.7	70.9
	Other cereal products	6.1	6.1	6.8	9.3
Vegetables		196.9	217.9	214.9	176.1
	Cabbages	7.6	11.3	11.5	12.2
	Mushrooms	1.6	2.0	2.0	3.1
	Root vegetables and tubers	37.4	55.8	58.8	85.5
	Fruit vegetables	101.7	100.0	94.2	32.1
	Leaf vegetables	21.0	27.6	28.3	32.9
	Onion-family vegetables	9.2	9.4	9.4	10.2
	Canned or other vegetable products	18.4	11.9	10.7	0.0
Starchy/Tubers		58.5	69.2	70.2	79.9
	Potatoes	57.3	68.3	69.2	78.7
	Potato products	1.2	0.9	1.0	1.2
Legumes & Nuts		24.5	36.6	38.7	51.5
	Legumes	16.7	28.2	29.5	39.9
	Legume products	0.4	0.4	0.4	0.4
	Soya and soya products	2.2	2.2	2.2	2.2
	Nuts and seeds	5.2	5.9	6.6	9.0
Fruits		169.9	209.0	206.3	154.6
	Berries	34.0	42.1	39.2	1.7
	Citrus fruits	31.8	59.3	61.0	65.4
	Miscellaneous fruits	57.2	64.8	62.0	25.2
	Pome fruits	37.5	37.7	39.6	62.3
	Canned or dried fruit	9.3	5.0	4.5	0.0
Meats		79.1	68.8	68.8	68.8
	Beef	13.3	12.8	0.0	0.0

Food category by group		Current	NUTR	NUTR+ GHGE-25%	NUTR+ GHGE-55%
	Pork	7.0	4.8	2.4	0.0
	Poultry	23.8	22.2	33.1	36.9
	Lamb	0.4	0.2	0.0	0.0
	Game	1.1	0.9	0.9	0.0
	Edible offal	1.4	1.4	1.0	0.0
	Meat cuts	15.8	13.3	14.4	0.7
	Sausage	16.2	7.2	7.2	0.0
	Mycoprotein	0.0	5.9	9.9	31.2
Fish/Seafood		27.0	31.9	28.6	11.3
	Fish	15.6	22.1	20.7	11.3
	Fish products	10.0	8.7	7.4	0.0
	Crustaceans and molluscs	1.5	1.2	0.5	0.0
Dairy		394.8	424.7	373.7	268.0
	Milk, fat >2%	10.9	6.6	6.8	7.1
	Milk, fat <=2%	95.1	95.1	91.4	86.9
	Skimmed milk	78.6	127.9	122.6	154.0
	Milk powder	0.5	0.4	0.2	0.0
	Yoghurt	58.2	52.8	42.8	0.0
	Quark	23.6	27.2	15.7	0.0
	Cream	12.2	6.7	3.8	0.0
	Soured and cultured milks	29.8	38.5	35.9	0.0
	Fermented milk products, other	3.2	3.2	0.0	0.0
	Cheese, ripened or processed > 17 %	20.7	10.8	9.8	5.5
	Cheese, ripened or processed <=17 %	5.7	3.7	2.8	0.0
	Cheese, unripened, fresh >15 %	6.7	3.4	0.0	0.0
	Cheese, unripened, fresh <=15 %	9.7	10.2	5.9	0.0
	Dairy dessert	24.4	15.3	13.2	0.0
	Dairy imitates	15.5	22.9	22.9	14.5
Eggs		20.3	24.6	25.2	23.2
Sugar		25.0	21.9	21.1	15.6
	Chocolate	7.2	6.0	3.3	0.0
	Non-chocolate confectionery	9.0	7.3	8.7	7.2
	Jams	4.4	4.8	5.0	3.6
	Sugar and syrups	4.4	3.8	4.0	4.9
Fats		30.3	27.0	28.5	34.7
	Vegetable fat and oil	2.2	1.4	1.5	1.6
	Margarine and similar products	6.2	3.6	4.3	8.0
	Salad dressings and mayonnaises	4.8	3.8	3.9	2.4
	Butter, milk fat	1.8	1.0	0.7	0.0
	Blended fat and oil	15.3	17.2	18.1	22.7
Water & Drinks		1982.2	1985.3	1924.4	1530.5
	Water	1221.4	1221.4	1221.4	1221.4

Food category by group	Current	NUTR	NUTR+ GHGE-25%	NUTR+ GHGE-55%
Soft drinks (incl. sport drinks)	55.0	49.4	40.8	0.0
Coffee beverages	409.2	400.7	345.0	0.0
Tea	149.3	133.8	156.1	207.4
Beer, cider and long drink	44.7	33.8	10.5	0.0
Wines and spirits	17.5	6.6	4.5	0.0
Juice	85.2	139.6	146.1	101.7
Spices & Condiments	17.5	14.2	14.4	11.7
Condiments	15.2	11.9	12.1	9.2
Dried spices and herbs	0.6	0.7	0.7	0.8
Miscellaneous ingredients	0.6	0.7	0.8	0.9
Salt	1.2	0.8	0.8	0.8

Supplemental Table 3: Food categorical intake (g/cap/day) of the observed diet and three optimized diets ("NUTR", "NUTR+GHGE-25%" and "NUTR+GHGE-55%") for an average Finnish male. The cells in yellow/green means that the categorical intake reaches the minimum/maximum feasible consumption constraint.

Food category by group	Current	NUTR	NUTR+ GHGE-25%	NUTR+ GHGE-55%
Grains	369.8	482.7	490.8	563.9
Rice and rice flour	60.6	91.2	85.0	0.0
Wheat and wheat flour	12.5	14.8	15.6	20.5
Rye and rye flour	0.2	0.2	0.2	0.2
Other grain and grain flour	0.9	0.9	0.9	0.9
Starch	1.4	1.3	1.3	1.4
Wheat bread and rolls	22.8	19.6	20.7	32.3
Rye bread and rolls	43.3	108.8	111.1	135.6
Multigrain bread and rolls	42.8	85.5	87.6	113.5
Pasta	33.0	37.4	38.3	33.4
Noodles	7.8	5.8	6.4	7.8
Breakfast cereal	90.8	75.4	77.7	146.6
Fine bakerly wares	49.3	37.0	40.9	65.2
Other cereal products	4.5	4.7	5.0	6.6
Vegetables	180.5	180.2	177.9	162.3
Cabbages	6.3	8.3	8.2	7.1
Mushrooms	2.0	2.3	2.4	2.9
Root vegetables and tubers	36.5	55.8	57.2	79.5
Fruit vegetables	83.7	74.1	71.6	40.7
Leaf vegetables	17.5	19.2	19.1	18.6
Onion-family vegetables	11.5	12.0	12.0	12.3
Canned or other vegetable products	23.1	8.6	7.4	1.2
Starchy/Tubers	85.6	99.5	99.6	104.9
Potatoes	82.6	97.4	97.3	101.2
Potato products	3.0	2.2	2.4	3.7
Legumes & Nuts	23.9	33.6	33.9	39.7
Legumes	17.3	25.0	25.1	29.6
Legume products	0.3	0.3	0.3	0.3
Soya and soya products	2.2	2.2	2.1	2.2

Food category by group	Current	NUTR	NUTR+ GHGE-25%	NUTR+ GHGE-55%
Nuts and seeds	4.1	6.1	6.5	7.6
Fruits	123.5	166.0	164.7	136.3
Berries	22.0	30.5	29.3	13.2
Citrus fruits	23.2	34.4	34.1	36.9
Miscellaneous fruits	42.4	53.2	52.2	30.3
Pome fruits	27.6	39.5	40.5	50.9
Canned or dried fruit	8.3	8.4	8.5	5.1
Meats	136.2	112.1	112.1	112.1
Beef	21.7	21.8	1.3	0.0
Pork	15.1	7.0	5.4	0.0
Poultry	30.1	30.8	41.6	47.8
Lamb	1.0	0.5	0.0	0.0
Game	2.6	0.3	0.6	0.0
Edible offal	2.8	2.8	2.7	0.9
Meat cuts	28.0	16.1	18.4	6.7
Sausage	34.7	5.3	9.6	0.0
Mycoprotein	0.0	27.3	32.5	56.7
Fish/Seafood	33.5	26.9	24.8	13.6
Fish	20.5	23.4	22.5	13.6
Fish products	11.9	2.5	1.4	0.0
Crustaceans and molluscs	1.1	1.0	0.9	0.0
Dairy	461.6	379.1	352.0	199.1
Milk, fat >2%	19.0	12.9	13.0	14.2
Milk, fat <=2%	152.9	138.9	135.0	116.8
Skimmed milk	94.6	78.9	71.6	45.9
Milk powder	1.2	0.8	0.6	0.0
Yoghurt	55.8	53.3	48.6	0.0
Quark	18.1	9.9	4.6	0.0
Cream	14.8	12.7	11.6	0.0
Soured and cultured milks	27.2	29.9	28.2	0.0
Fermented milk products, other	1.2	1.2	0.9	0.0
Cheese, ripened or processed > 17 %	31.4	16.3	15.6	18.4
Cheese, ripened or processed <=17 %	5.5	1.1	0.6	0.0

Food category by group	Current	NUTR	NUTR+ GHGE-25%	NUTR+ GHGE-55%
Cheese, unripened, fresh >15 %	4.2	2.5	1.7	0.0
Cheese, unripened, fresh <=15 %	6.9	0.0	0.0	0.0
Dairy dessert	18.0	14.2	13.7	0.0
Dairy imitates	10.8	6.4	6.2	3.7
Eggs	21.3	18.6	18.6	19.6
Sugar	24.8	24.8	24.6	21.1
Chocolate	6.7	7.7	6.6	0.0
Non-chocolate confectionery	7.6	6.7	7.3	8.8
Jams	4.4	4.7	4.8	4.6
Sugar and syrups	6.2	5.7	6.0	7.7
Fats	42.8	36.3	37.8	49.0
Vegetable fat and oil	2.6	2.5	2.6	2.7
Margarine and similar products	10.1	7.6	8.6	17.7
Salad dressings and mayonnaises	6.6	6.0	6.0	5.5
Butter, milk fat	2.8	1.7	1.3	0.0
Blended fat and oil	20.6	18.5	19.4	23.1
Water & Drinks	2034.1	1971.7	1925.2	1431.6
Water	1047.3	1047.4	1047.4	1047.4
Soft drinks (incl. sport drinks)	99.8	83.5	79.4	0.0
Coffee beverages	530.6	545.4	524.8	209.1
Tea	86.1	77.7	82.5	110.2
Beer, cider and long drink	138.9	91.3	64.5	0.0
Wines and spirits	16.2	11.2	11.6	0.0
Juice	115.1	115.3	115.0	64.9
Spices & Condiments	25.7	14.3	14.1	12.6
Condiments	22.9	13.2	13.0	11.5
Dried spices and herbs	0.4	0.4	0.4	0.4
Miscellaneous ingredients	0.9	0.5	0.5	0.5
Salt	1.5	0.2	0.2	0.1

Supplemental Figures

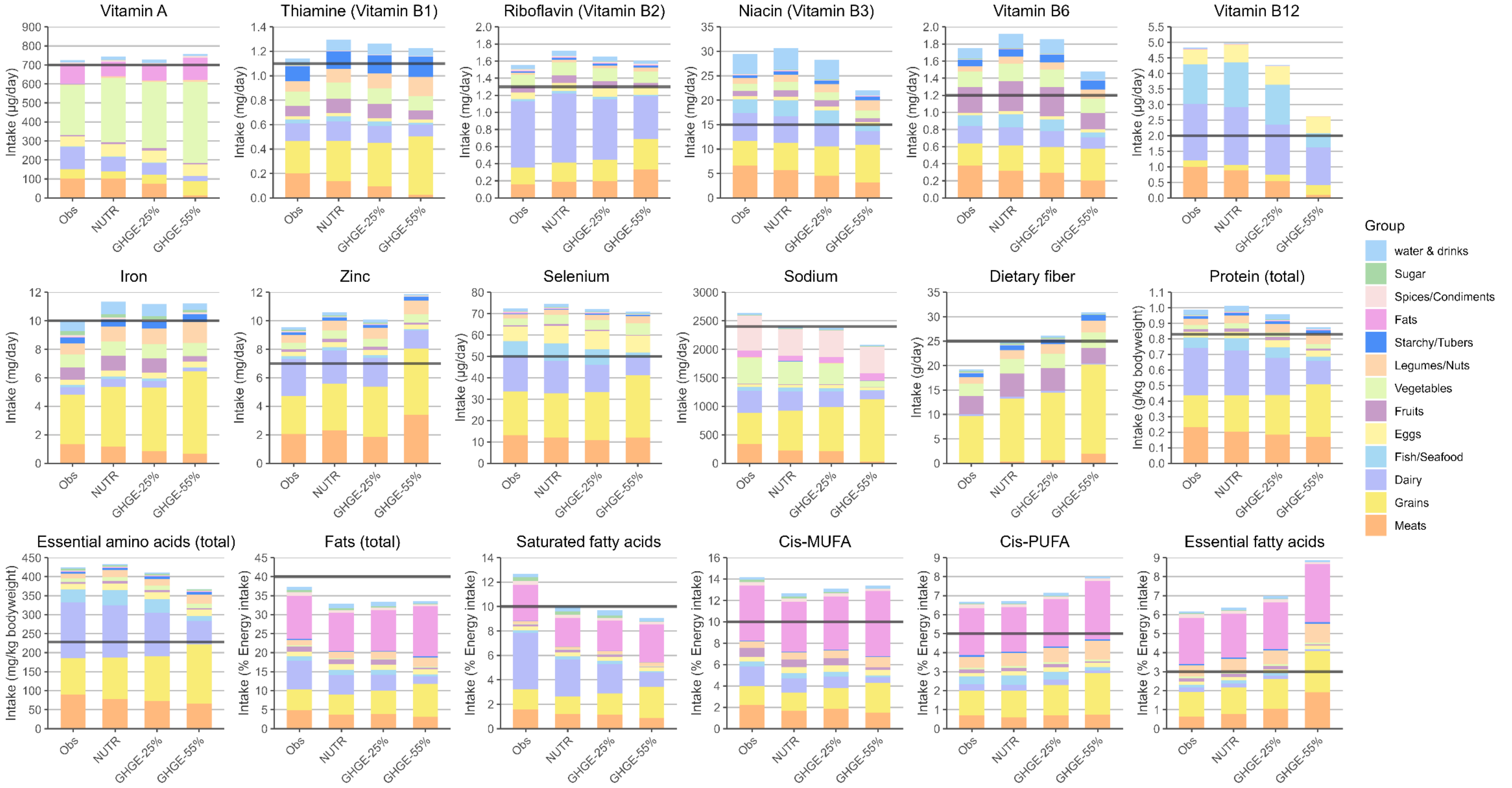

Supplemental Figure 1: Nutritional contributions of food groups to total nutrient intakes, for selected nutrients (Female). The solid line represents the constraint for each nutrient (lower bound, except for sodium, fats (total) and saturated fatty acids that are upper bounds).

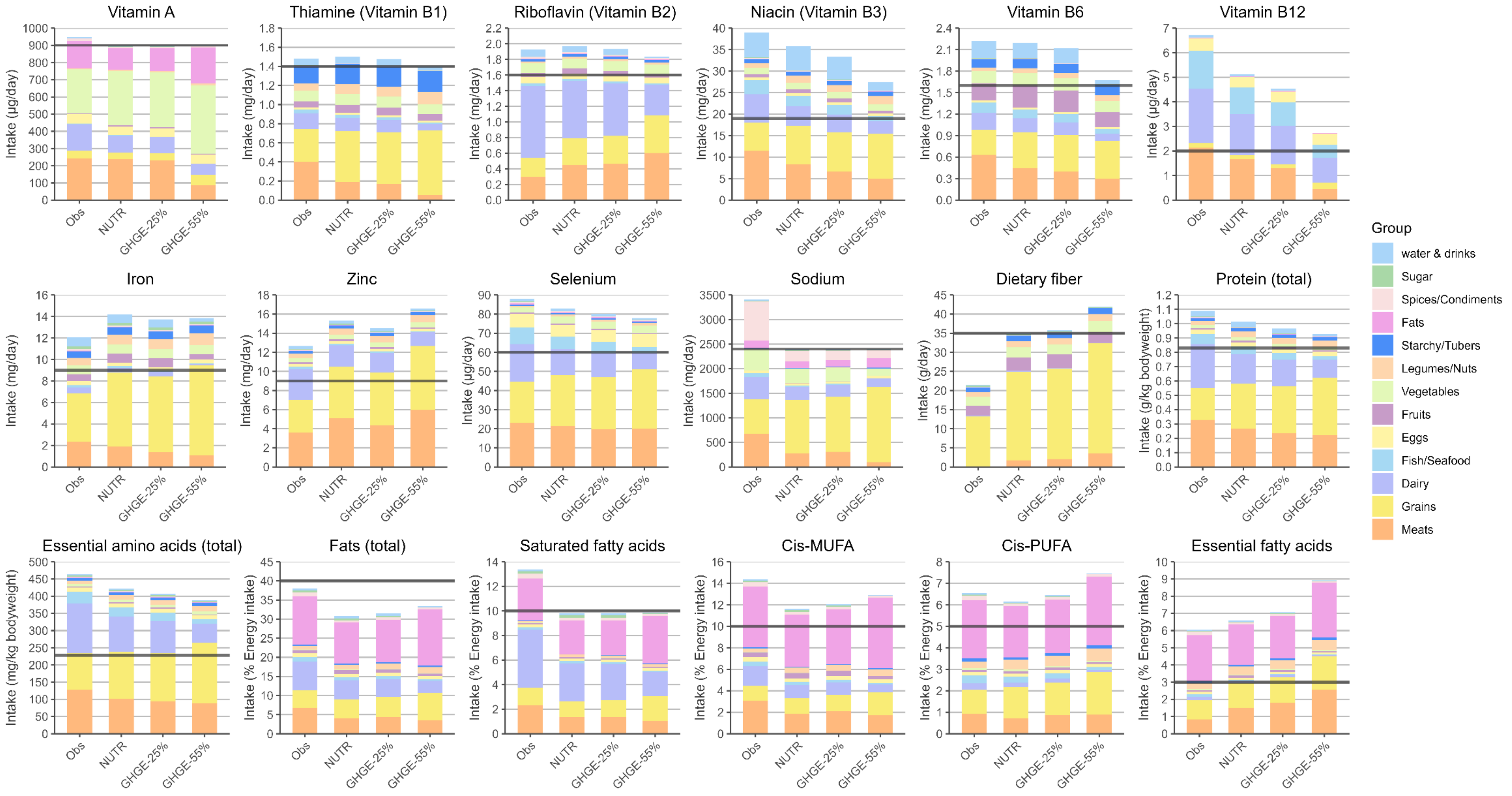

Supplemental Figure 2: Nutritional contributions of food groups to total nutrient intakes, for selected nutrients (Male). The solid line represents the constraint for each nutrient (lower bound, except for sodium, fats (total) and saturated fatty acids that are upper bounds).

Female			Dietary intake (g/cap/day)						Nutrient composition (per 100g)		
#	Food group	Food category	Mean	5th percentile	95th percentile	Standard Deviation	Price (€/kg)	Global Warming Potential (kg CO2eq/kg)	Vitamin A (µg)	Vitamin D (µg)	Vitamin E (mg)
	group	category	q0	P5	P95	sd	price	lca	VITA	VITD	VITE
1	Dairy	Cheese, ripened or processed <=17 %	5.690	0.000	34.966	10.749	9.056	6.664	93.016	0.094	0.206
2	Dairy	Cheese, ripened or processed > 17 %	20.747	0.000	120.070	21.779	9.016	6.664	228.211	0.228	0.555
3	Dairy	Cheese, unripened, fresh cheese <=15 %	9.671	0.000	56.750	26.041	8.466	5.816	20.840	0.016	0.070
4	Dairy	Cheese, unripened, fresh cheese >15 %	6.688	0.000	15.671	23.946	9.013	5.801	90.916	0.061	0.151
5	Dairy	Cream	12.193	0.000	57.217	19.755	5.080	7.476	184.087	0.251	0.533
6	Dairy	Dairy dessert	24.420	0.000	83.527	47.164	5.084	2.924	32.458	0.447	0.278
7	Dairy	Dairy imitates	15.452	0.000	22.859	48.232	2.122	1.897	0.187	0.943	0.418
8	Dairy	Fermented milk products, other	3.242	0.000	3.242	61.108	4.310	3.769	2.296	0.094	0.160
9	Dairy	Milk powder	0.494	0.000	0.494	2.720	10.520	15.791	6.309	0.554	0.009
10	Dairy	Milk, fat <=2%	95.091	0.000	375.232	141.471	1.009	1.564	14.356	1.009	0.030
11	Dairy	Milk, fat >2%	10.884	0.000	37.500	54.546	0.836	1.564	29.588	0.782	0.069
12	Dairy	Quark	23.648	0.000	141.185	60.805	8.466	3.784	1.480	0.020	0.010
13	Dairy	Skimmed milk	78.580	0.000	436.471	158.066	1.011	1.564	0.787	1.063	0.001
14	Dairy	Soured and cultured milks	29.841	0.000	270.000	78.323	1.842	1.790	2.626	0.027	0.070
15	Dairy	Yoghurt	58.201	0.000	285.757	82.934	2.328	2.871	11.762	0.405	0.062
16	Eggs	Eggs	20.258	0.000	136.690	31.055	3.837	2.575	257.207	2.331	2.254
17	Fats	Blended fat and oil	15.286	0.000	68.952	16.339	5.722	5.438	174.799	17.647	12.803
18	Fats	Butter, milk fat	1.786	0.000	8.460	3.851	4.930	15.594	710.489	0.327	1.520
19	Fats	Margarine and similar products	6.172	0.000	38.042	11.344	4.210	1.645	900.000	7.500	8.320
20	Fats	Salad dressings and mayonnaises	4.840	0.000	28.697	8.401	5.000	2.550	171.975	0.430	8.784
21	Fats	Vegetable fat and oil	2.232	0.000	13.500	4.598	5.358	2.146	0.902	0.000	15.906
22	Fish/Seafood	Crustaceans and molluscs	1.457	0.000	1.192	8.877	18.449	7.382	4.052	0.041	3.507
23	Fish/Seafood	Fish	15.600	0.000	62.951	30.766	10.514	6.582	8.844	7.634	1.668
24	Fish/Seafood	Fish products	9.967	0.000	47.506	21.051	15.130	7.323	16.706	6.854	1.538
25	Fruits	Berries	34.045	0.000	208.727	41.426	4.107	2.510	3.504	0.000	1.280
26	Fruits	Canned or dried fruit	9.273	0.000	46.648	24.042	5.681	2.670	15.795	0.000	2.014
27	Fruits	Citrus fruits	31.818	0.000	183.251	63.890	2.437	1.113	9.936	0.000	0.348
28	Fruits	Miscellaneous fruits	57.223	0.000	278.572	68.848	2.791	2.145	5.066	0.000	0.532
29	Fruits	Pome fruits	37.499	0.000	226.859	65.248	1.950	0.409	1.450	0.000	0.215
30	Grains	Breakfast cereal	77.624	0.000	400.542	109.272	0.218	0.154	1.508	0.110	0.188
31	Grains	Fine bakery wares	47.142	0.000	280.978	52.277	8.233	1.533	96.477	0.875	1.284
32	Grains	Multigrain bread and rolls	33.155	0.000	156.983	38.394	4.881	0.614	1.562	0.000	0.923
33	Grains	Noodles	8.289	0.000	10.064	44.558	1.838	0.321	0.119	0.000	0.158
34	Grains	Other cereal products	6.110	0.000	32.460	12.291	6.816	1.704	4.626	0.002	2.655
35	Grains	Other grain and grain flour	1.176	0.000	1.176	6.788	3.684	1.520	0.212	0.000	0.761
36	Grains	Pasta	20.535	0.000	91.300	46.305	1.838	0.596	0.356	0.002	0.149
37	Grains	Rice and rice flour	41.694	0.000	276.799	87.048	1.162	2.283	0.000	0.000	0.136
38	Grains	Rye and rye flour	0.143	0.000	0.143	1.022	1.000	1.051	0.521	0.000	0.934
39	Grains	Rye bread and rolls	27.195	0.000	105.447	34.101	4.240	0.704	0.324	0.000	0.716
40	Grains	Starch	1.030	0.000	6.277	2.360	2.860	1.704	0.030	0.000	0.026
41	Grains	Wheat and wheat flour	8.369	0.000	40.865	15.041	0.761	0.596	0.364	0.000	0.442
42	Grains	Wheat bread and rolls	13.885	0.000	86.092	23.215	4.300	0.415	2.883	0.034	0.733
43	Legumes/Nuts	Legume products	0.398	0.000	0.398	3.909	3.554	1.028	0.296	0.000	5.021
44	Legumes/Nuts	Legumes	16.651	0.000	39.935	34.080	3.275	0.458	28.157	0.000	0.063
45	Legumes/Nuts	Nuts and seeds	5.221	0.000	22.334	10.750	13.423	2.511	1.260	0.000	8.858
46	Legumes/Nuts	Soya and soya products	2.195	0.000	2.195	8.938	2.473	3.998	3.675	0.000	1.035
47	Meats	Beef	13.304	0.000	70.295	19.340	14.552	58.974	12.173	0.285	0.580
48	Meats	Edible offal	1.403	0.000	1.403	7.429	7.492	27.205	6386.464	0.433	0.524
49	Meats	Game	1.124	0.000	1.124	6.845	13.463	9.178	7.340	0.253	1.011
50	Meats	Lamb	0.396	0.000	0.396	3.581	15.668	55.688	12.774	0.239	0.382
51	Meats	Meat cuts	15.792	0.000	82.102	21.377	9.424	8.121	5.482	0.315	0.436
52	Meats	Pork	6.999	0.000	35.829	13.700	12.123	18.771	12.011	0.579	0.528
53	Meats	Poultry	23.837	0.000	93.090	34.071	6.153	6.311	36.211	0.808	0.814
54	Meats	Sausage	16.214	0.000	79.806	29.954	7.108	10.768	8.241	0.223	0.344
55	Meats	Mycoprotein	0.000	0.000	284.977	23.689	13.149	2.291	0.000	0.000	0.000
56	Spices/Condiments	Condiments	15.167	0.000	101.783	30.464	6.186	1.411	56.393	0.240	1.489
57	Spices/Condiments	Dried spices and herbs	0.578	0.000	1.901	2.193	25.144	1.411	319.449	0.000	2.083
58	Spices/Condiments	Miscellaneous ingredients	0.610	0.000	4.470	1.230	8.081	0.000	0.000	0.000	0.564
59	Spices/Condiments	Salt	1.169	0.000	3.227	0.889	2.180	0.000	0.000	0.000	0.000
60	Starchy/Tubers	Potato products	1.246	0.000	1.246	5.900	3.847	1.733	5.594	0.002	4.229
61	Starchy/Tubers	Potatoes	57.259	0.000	164.910	56.537	0.830	1.122	0.638	0.000	0.050
62	Sugar	Chocolate	7.176	0.000	55.408	13.459	15.080	17.469	5.220	0.003	0.673
63	Sugar	Jams	4.399	0.000	16.299	11.985	7.119	2.067	7.854	0.000	0.396
64	Sugar	Non-chocolate confectionery	8.969	0.000	43.449	18.600	13.832	3.234	0.081	0.000	0.139
65	Sugar	Sugar and syrups	4.423	0.000	21.521	7.330	1.365	0.803	0.000	0.000	0.000
66	Vegetables	Cabbages	7.649	0.000	45.208	23.097	2.310	1.297	4.851	0.000	0.059
67	Vegetables	Canned or other vegetable products	18.390	0.000	88.643	28.172	4.180	2.278	176.332	0.020	0.973
68	Vegetables	Fruit vegetables	101.661	0.000	320.886	71.743	2.608	2.066	43.583	0.000	0.501
69	Vegetables	Leaf vegetables	20.960	0.000	89.742	22.048	7.322	1.195	71.103	0.000	0.780
70	Vegetables	Mushrooms	1.589	0.000	7.272	6.460	4.569	0.121	21.658	4.294	0.000
71	Vegetables	Onion-family vegetables	9.221	0.000	29.120	9.525	1.573	0.542	0.567	0.000	0.041
72	Vegetables	Root vegetables and tubers	37.414	0.000	162.414	39.362	1.806	0.506	452.605	0.000	0.340
73	water & drinks	Beer, cider and long drink	44.725	0.000	165.000	140.516	4.769	1.269	0.010	0.000	0.002
74	water & drinks	Coffee beverages	409.213	0.000	1177.408	328.647	0.454	0.603	0.258	0.008	0.001
75	water & drinks	Juice	85.160	0.000	494.217	127.777	1.565	0.936	11.997	0.003	0.384
76	water & drinks	Soft drinks (incl. sport drinks)	54.966	0.000	301.146	120.482	1.765	0.711	0.198	0.000	0.022
77	water & drinks	Tea	149.280	0.000	910.256	281.968	0.083	0.102	0.005	0.000	0.001
78	water & drinks	Water	1221.381	1221.381	1221.381	766.897	0.000	0.000	0.000	0.000	0.006
79	water & drinks	Wines and spirits	17.486	0.000	150.844	52.467	11.923	1.276	0.622	0.000	0.002

Female			Nutrient composition (per 100g)									
#	Food group	Food category	Thiamin/ B1 (mg)	Riboflavin/ B2 (mg)	Niacin/B3 (mg)	Vitamin B6 (mg)	Folate (µg)	Vitamin B12 (µg)	Vitamin C (mg)	Calcium (mg)	Phosphorus (mg)	Magnesium (mg)
	group	category	THIA	RIBF	NIAEQ	VITPYRID	FOL	VITB12	VITC	CA	P	MG
1	Dairy	Cheese, ripened or processed <=17 %	0.027	0.240	3.034	0.044	17.469	0.622	0.281	531.182	334.285	19.452
2	Dairy	Cheese, ripened or processed > 17 %	0.026	0.328	6.046	0.054	16.756	1.227	0.052	842.846	489.474	30.556
3	Dairy	Cheese, unripened, fresh cheese <=15 %	0.030	0.224	3.804	0.060	27.000	0.700	0.000	70.000	200.000	9.000
4	Dairy	Cheese, unripened, fresh cheese >15 %	0.055	0.201	3.252	0.056	13.526	0.592	0.273	171.100	168.460	11.938
5	Dairy	Cream	0.036	0.147	0.522	0.029	7.305	0.396	0.845	84.100	67.870	9.050
6	Dairy	Dairy dessert	0.040	0.217	1.012	0.088	5.984	0.515	1.500	124.241	49.833	9.318
7	Dairy	Dairy imitates	0.059	0.153	0.881	0.028	19.665	0.360	0.141	118.936	60.157	19.354
8	Dairy	Fermented milk products, other	0.108	0.046	1.019	0.049	33.105	0.034	0.450	118.944	57.145	23.965
9	Dairy	Milk powder	0.350	2.194	9.416	0.479	20.541	2.478	2.478	1324.993	967.103	127.474
10	Dairy	Milk, fat <=2%	0.035	0.187	0.906	0.050	4.240	0.438	1.088	127.500	90.000	11.000
11	Dairy	Milk, fat >2%	0.033	0.183	0.842	0.050	5.827	0.399	0.980	123.140	90.230	11.285
12	Dairy	Quark	0.046	0.226	2.772	0.050	16.000	0.600	1.000	117.000	180.000	12.000
13	Dairy	Skimmed milk	0.034	0.187	0.980	0.050	7.444	0.436	1.225	140.150	89.725	14.250
14	Dairy	Soured and cultured milks	0.034	0.153	0.873	0.043	12.289	0.308	0.770	95.928	89.626	12.947
15	Dairy	Yoghurt	0.034	0.180	0.844	0.051	5.053	0.249	1.756	122.132	90.827	11.875
16	Eggs	Eggs	0.117	0.378	3.203	0.141	58.963	2.324	0.000	58.313	212.819	13.229
17	Fats	Blended fat and oil	0.001	0.013	0.055	0.000	0.694	0.000	0.023	11.001	9.190	1.846
18	Fats	Butter, milk fat	0.000	0.030	0.101	0.000	3.018	0.000	0.000	23.838	33.865	2.584
19	Fats	Margarine and similar products	0.000	0.000	0.073	0.000	2.000	0.000	0.000	11.000	10.000	2.200
20	Fats	Salad dressings and mayonnaises	0.026	0.096	0.445	0.030	10.344	0.414	0.618	47.295	66.100	6.776
21	Fats	Vegetable fat and oil	0.000	0.000	0.000	0.000	0.000	0.000	0.000	0.000	0.000	0.000
22	Fish/Seafood	Crustaceans and molluscs	0.021	0.039	6.502	0.336	21.936	5.962	0.000	53.455	120.909	23.454
23	Fish/Seafood	Fish	0.141	0.105	9.579	0.523	13.085	4.034	0.000	40.745	213.104	23.495
24	Fish/Seafood	Fish products	0.100	0.105	12.134	0.392	8.222	5.554	0.034	28.125	192.702	26.831
25	Fruits	Berries	0.080	0.025	0.560	0.056	15.070	0.000	22.225	20.846	25.741	11.587
26	Fruits	Canned or dried fruit	0.044	0.036	0.554	0.104	3.629	0.000	7.159	18.234	20.635	16.230
27	Fruits	Citrus fruits	0.082	0.036	0.525	0.087	25.152	0.000	47.828	49.004	21.281	12.880
28	Fruits	Miscellaneous fruits	0.040	0.072	0.924	0.390	12.743	0.000	13.371	9.540	24.678	26.643
29	Fruits	Pome fruits	0.015	0.017	0.221	0.054	2.766	0.000	7.720	5.839	9.559	4.849
30	Grains	Breakfast cereal	0.061	0.044	1.033	0.051	7.460	0.049	0.130	22.445	71.353	22.661
31	Grains	Fine bakerly wares	0.052	0.131	1.793	0.073	22.118	0.358	1.663	99.590	139.027	19.194
32	Grains	Multigrain bread and rolls	0.184	0.099	2.394	0.119	33.201	0.000	0.273	25.807	196.201	65.519
33	Grains	Noodles	0.032	0.019	0.886	0.019	3.679	0.000	0.000	11.869	55.249	9.140
34	Grains	Other cereal products	0.300	0.113	3.776	0.275	33.219	0.002	0.314	60.541	302.843	124.575
35	Grains	Other grain and grain flour	0.440	0.115	4.658	0.154	33.264	0.000	0.000	40.177	362.934	119.991
36	Grains	Pasta	0.017	0.011	1.140	0.086	4.726	0.003	0.000	6.469	49.632	17.565
37	Grains	Rice and rice flour	0.051	0.013	1.689	0.135	4.730	0.000	0.000	5.610	87.845	35.307
38	Grains	Rye and rye flour	0.332	0.156	2.836	0.165	38.261	0.000	0.000	30.576	311.517	100.588
39	Grains	Rye bread and rolls	0.182	0.123	1.984	0.123	36.302	0.000	0.053	26.160	215.065	68.920
40	Grains	Starch	0.004	0.003	0.182	0.011	17.341	0.000	0.003	22.246	60.145	6.756
41	Grains	Wheat and wheat flour	0.098	0.055	3.438	0.113	19.089	0.000	0.000	20.042	138.026	38.218
42	Grains	Wheat bread and rolls	0.168	0.084	3.518	0.086	29.077	0.004	0.009	23.796	121.517	32.158
43	Legumes/Nuts	Legume products	0.603	0.134	10.331	0.219	103.369	0.000	1.071	140.012	304.000	151.397
44	Legumes/Nuts	Legumes	0.187	0.044	2.640	0.166	50.499	0.000	14.547	39.109	128.009	42.159
45	Legumes/Nuts	Nuts and seeds	0.801	0.230	9.211	0.402	75.149	0.000	0.560	137.104	501.951	261.742
46	Legumes/Nuts	Soya and soya products	0.439	0.193	8.920	0.418	181.831	0.008	0.597	319.017	442.514	187.429
47	Meats	Beef	0.135	0.226	14.063	0.591	5.404	1.971	0.000	9.679	245.189	29.244
48	Meats	Edible offal	0.365	1.271	10.449	0.517	437.457	26.015	9.837	14.782	255.855	23.900
49	Meats	Game	0.145	0.290	10.080	0.728	6.468	6.389	0.000	13.158	332.290	38.069
50	Meats	Lamb	0.138	0.290	7.928	0.270	2.030	0.950	0.000	11.040	227.300	27.764
51	Meats	Meat cuts	0.147	0.192	9.000	0.461	3.756	0.567	0.000	7.787	173.054	14.932
52	Meats	Pork	1.255	0.353	6.836	0.490	2.250	0.870	0.000	12.410	224.000	26.995
53	Meats	Poultry	0.037	0.140	7.155	0.550	7.375	0.234	0.000	12.780	209.007	19.767
54	Meats	Sausage	0.332	0.120	5.505	0.261	3.821	0.563	0.064	19.596	117.878	17.073
55	Meats	Mycoprotein	0.040	0.900	1.400	0.000	0.000	0.000	0.000	42.500	260.000	6.000
56	Spices/Condiments	Condiments	0.015	0.046	0.619	0.030	4.561	0.051	2.511	27.579	35.387	7.470
57	Spices/Condiments	Dried spices and herbs	0.085	0.256	1.885	0.185	110.278	0.000	91.046	214.968	68.410	41.460
58	Spices/Condiments	Miscellaneous ingredients	0.268	1.210	13.771	0.257	668.806	0.019	0.000	34.318	381.846	71.846
59	Spices/Condiments	Salt	0.000	0.000	0.000	0.000	0.000	0.000	0.000	157.275	70.060	45.161
60	Starchy/Tubers	Potato products	0.186	0.061	4.139	0.523	35.695	0.000	3.492	24.715	134.873	49.218
61	Starchy/Tubers	Potatoes	0.210	0.030	0.916	0.122	23.106	0.000	9.994	5.596	45.036	23.984
62	Sugar	Chocolate	0.007	0.047	0.575	0.026	3.564	0.035	0.118	30.434	73.215	43.652
63	Sugar	Jams	0.020	0.023	0.372	0.052	4.863	0.000	5.786	11.293	13.697	9.267
64	Sugar	Non-chocolate confectionery	0.016	0.013	0.434	0.005	1.051	0.004	0.015	128.632	31.571	48.714
65	Sugar	Sugar and syrups	0.002	0.010	0.047	0.000	0.000	0.000	0.940	5.502	1.825	2.635
66	Vegetables	Cabbages	0.069	0.049	0.728	0.158	32.475	0.000	35.400	37.223	28.048	10.014
67	Vegetables	Canned or other vegetable products	0.109	0.101	0.821	0.087	17.999	0.000	9.889	20.236	32.066	13.561
68	Vegetables	Fruit vegetables	0.048	0.058	0.639	0.090	13.860	0.000	24.150	10.708	23.966	9.661
69	Vegetables	Leaf vegetables	0.053	0.091	0.654	0.078	63.128	0.000	11.763	62.093	40.074	23.116
70	Vegetables	Mushrooms	0.078	0.294	5.807	0.189	20.843	0.007	3.406	5.195	64.475	8.590
71	Vegetables	Onion-family vegetables	0.032	0.020	0.437	0.153	13.306	0.000	10.339	32.694	42.304	10.939
72	Vegetables	Root vegetables and tubers	0.079	0.078	1.013	0.085	44.621	0.000	20.119	29.909	45.540	15.125
73	water & drinks	Beer, cider and long drink	0.006	0.013	0.239	0.013	4.235	0.000	0.359	5.863	12.978	7.043
74	water & drinks	Coffee beverages	0.000	0.005	0.671	0.000	0.060	0.003	0.007	4.589	8.823	10.557
75	water & drinks	Juice	0.062	0.032	0.986	0.095	19.980	0.000	28.919	13.169	18.001	11.355
76	water & drinks	Soft drinks (incl. sport drinks)	0.007	0.020	0.538	0.063	2.248	0.055	4.309	3.756	6.261	3.669
77	water & drinks	Tea	0.002	0.008	0.001	0.000	1.724	0.000	1.051	0.558	1.333	0.287
78	water & drinks	Water	0.000	0.000	0.000	0.000	0.000	0.000	0.000	3.014	0.001	0.058
79	water & drinks	Wines and spirits	0.004	0.014	0.123	0.029	0.367	0.000	0.000	8.450	7.423	9.240

Supplemental Data 1: data for different sustainability metrics of foods by category (female)

Female			Nutrient composition (per 100g)									
#	Food group	Food category	Potassium (g)	Iron (mg)	Zinc (mg)	Iodine (µg)	Selenium (µg)	Copper (mg)	Sodium (mg)	NACL (g)	Saturated fatty acids (g)	Total fat (g)
	group	category	K	FE	ZN	ID	SE	CU	Sodium	NACL	fasatra	FAT
1	Dairy	Cheese, ripened or processed <=17 %	0.253	0.195	1.892	22.490	15.317	0.153	1033.846	2.634	6.619	10.644
2	Dairy	Cheese, ripened or processed > 17 %	0.084	0.280	3.693	26.789	18.921	0.327	531.858	1.355	19.272	29.844
3	Dairy	Cheese, unripened, fresh cheese <=15 %	0.100	0.120	1.350	17.000	12.218	0.034	300.000	0.764	1.371	2.000
4	Dairy	Cheese, unripened, fresh cheese >15 %	0.149	0.176	0.872	18.776	4.948	0.044	133.191	0.339	5.466	8.564
5	Dairy	Cream	0.121	0.127	0.356	14.034	2.480	0.010	101.903	0.260	14.644	22.224
6	Dairy	Dairy dessert	0.164	0.189	0.492	16.195	4.582	0.014	54.729	0.139	2.489	5.189
7	Dairy	Dairy imitates	0.129	0.647	0.292	4.789	1.538	0.089	89.200	0.227	1.226	4.701
8	Dairy	Fermented milk products, other	0.174	0.854	0.195	2.236	2.092	0.147	44.692	0.114	0.408	1.908
9	Dairy	Milk powder	1.727	0.478	4.523	144.328	19.447	0.096	292.997	0.747	2.468	3.915
10	Dairy	Milk, fat <=2%	0.151	0.050	0.410	14.725	2.750	0.009	41.000	0.104	1.003	1.438
11	Dairy	Milk, fat >2%	0.160	0.067	0.444	15.154	2.871	0.011	45.220	0.115	2.284	3.523
12	Dairy	Quark	0.170	0.050	0.550	17.000	3.853	0.026	99.140	0.253	0.126	0.400
13	Dairy	Skimmed milk	0.166	0.040	0.428	14.738	2.740	0.010	45.935	0.117	0.068	0.100
14	Dairy	Soured and cultured milks	0.142	0.317	0.457	13.596	2.507	0.021	38.435	0.098	0.140	0.569
15	Dairy	Yoghurt	0.170	0.070	0.433	14.826	2.640	0.012	44.442	0.113	0.804	1.286
16	Eggs	Eggs	0.131	1.725	1.420	49.450	33.380	0.051	218.856	0.558	2.833	11.395
17	Fats	Blended fat and oil	0.010	0.034	0.033	4.589	0.171	0.004	346.596	0.883	13.947	75.701
18	Fats	Butter, milk fat	0.018	0.181	0.080	4.159	0.503	0.011	576.311	1.468	53.092	81.804
19	Fats	Margarine and similar products	0.021	0.120	0.050	0.000	0.070	0.012	500.000	1.274	31.206	80.000
20	Fats	Salad dressings and mayonnaises	0.065	0.443	0.438	27.967	6.413	0.020	331.699	0.845	12.837	53.813
21	Fats	Vegetable fat and oil	0.000	0.003	0.000	0.000	0.000	0.000	0.000	0.000	12.752	99.999
22	Fish/Seafood	Crustaceans and molluscs	0.099	3.164	1.381	159.374	34.549	0.269	197.639	0.504	0.174	0.878
23	Fish/Seafood	Fish	0.358	0.415	0.586	52.868	23.077	0.057	45.480	0.116	1.795	9.114
24	Fish/Seafood	Fish products	0.332	0.654	0.977	31.472	30.644	0.069	611.476	1.558	1.398	9.432
25	Fruits	Berries	0.166	0.733	0.154	0.995	0.944	0.071	2.444	0.006	0.035	0.691
26	Fruits	Canned or dried fruit	0.196	0.497	0.239	0.937	0.213	0.094	2.885	0.007	1.567	11.118
27	Fruits	Citrus fruits	0.151	0.174	0.107	0.640	0.055	0.063	1.630	0.004	0.026	0.184
28	Fruits	Miscellaneous fruits	0.332	0.839	0.148	0.951	0.355	0.123	4.620	0.012	0.368	2.697
29	Fruits	Pome fruits	0.114	0.114	0.033	1.129	0.183	0.045	0.981	0.002	0.021	0.098
30	Grains	Breakfast cereal	0.088	0.784	0.559	13.205	1.840	0.083	158.287	0.403	0.235	1.136
31	Grains	Fine bakerly wares	0.152	0.715	0.694	17.279	8.427	0.084	189.473	0.483	5.002	12.087
32	Grains	Multigrain bread and rolls	0.275	2.145	1.717	18.254	4.557	0.266	432.354	1.102	0.525	3.274
33	Grains	Noodles	0.060	1.218	0.410	1.905	3.685	0.067	59.021	0.150	0.319	0.963
34	Grains	Other cereal products	0.440	2.780	2.481	14.945	118.403	0.728	425.836	1.085	4.914	29.533
35	Grains	Other grain and grain flour	0.370	4.133	2.744	13.702	8.604	0.422	1.720	0.004	0.631	3.984
36	Grains	Pasta	0.063	1.199	0.392	1.576	4.060	0.068	5.784	0.015	0.101	0.603
37	Grains	Rice and rice flour	0.086	0.846	0.609	1.747	5.113	0.109	2.610	0.007	0.161	0.760
38	Grains	Rye and rye flour	0.433	3.493	2.973	5.475	5.232	0.381	1.237	0.003	0.258	2.041
39	Grains	Rye bread and rolls	0.312	2.325	2.025	8.630	3.983	0.267	392.242	0.999	0.178	1.464
40	Grains	Starch	0.053	0.554	0.158	21.059	0.911	0.059	9.109	0.023	0.290	0.638
41	Grains	Wheat and wheat flour	0.180	1.137	0.980	9.860	10.026	0.197	1.042	0.003	0.212	1.611
42	Grains	Wheat bread and rolls	0.156	1.261	0.990	14.301	6.991	0.176	370.582	0.944	0.771	3.033
43	Legumes/Nuts	Legume products	0.580	3.709	2.362	4.016	8.176	0.599	156.493	0.399	3.620	26.156
44	Legumes/Nuts	Legumes	0.406	1.971	1.276	1.131	1.213	0.215	3.501	0.009	0.171	0.919
45	Legumes/Nuts	Nuts and seeds	0.707	5.295	4.407	5.673	29.690	1.446	10.378	0.026	5.878	49.067
46	Legumes/Nuts	Soya and soya products	1.449	8.142	3.168	3.460	7.960	1.284	123.141	0.314	0.726	6.647
47	Meats	Beef	0.451	3.459	6.900	4.966	23.242	0.112	85.190	0.217	2.579	5.965
48	Meats	Edible offal	0.299	9.050	3.421	9.295	49.079	1.579	597.748	1.523	5.013	13.533
49	Meats	Game	0.524	6.318	5.951	29.790	20.276	0.437	468.932	1.195	1.832	4.682
50	Meats	Lamb	0.382	2.871	4.414	28.620	16.107	0.121	463.960	1.182	6.811	14.909
51	Meats	Meat cuts	0.237	0.642	1.384	9.245	12.654	0.051	774.522	1.973	2.290	6.679
52	Meats	Pork	0.382	1.096	2.855	31.410	24.677	0.118	510.420	1.301	6.600	18.935
53	Meats	Poultry	0.210	1.391	1.525	17.352	15.223	0.069	69.516	0.177	3.389	13.855
54	Meats	Sausage	0.204	0.918	1.402	13.748	10.429	0.056	822.664	2.096	6.715	17.618
55	Meats	Mycoprotein	0.100	0.500	9.000	0.000	20.000	0.500	5.000	0.013	1.500	3.250
56	Spices/Condiments	Condiments	0.070	0.444	0.185	20.982	2.394	0.028	712.787	1.816	3.857	11.566
57	Spices/Condiments	Dried spices and herbs	0.590	4.580	1.023	3.789	3.063	0.216	21.767	0.055	0.078	0.634
58	Spices/Condiments	Miscellaneous ingredients	1.133	2.472	3.245	2.140	0.813	0.141	8836.505	22.515	4.564	9.978
59	Spices/Condiments	Salt	0.073	0.762	0.080	2266.407	0.455	0.182	38664.443	98.517	0.000	0.000
60	Starchy/Tubers	Potato products	1.042	1.568	0.697	0.698	3.268	0.193	493.792	1.258	4.092	27.220
61	Starchy/Tubers	Potatoes	0.500	0.670	0.310	1.000	0.637	0.091	1.000	0.003	0.025	0.114
62	Sugar	Chocolate	0.236	1.071	0.620	4.694	0.790	0.007	24.350	0.062	7.750	15.959
63	Sugar	Jams	0.117	0.327	0.120	2.189	0.427	0.047	1.614	0.004	0.047	0.243
64	Sugar	Non-chocolate confectionery	0.160	2.032	0.427	10.460	1.394	0.166	122.105	0.311	0.779	1.945
65	Sugar	Sugar and syrups	0.028	0.296	0.038	4.973	0.521	0.027	3.329	0.008	0.000	0.000
66	Vegetables	Cabbages	0.246	0.209	0.119	0.906	10.000	0.011	5.665	0.014	0.024	0.100
67	Vegetables	Canned or other vegetable products	0.267	0.545	0.194	14.116	0.753	0.081	1038.643	2.646	0.317	0.938
68	Vegetables	Fruit vegetables	0.192	0.350	0.127	0.968	0.414	0.038	256.873	0.654	0.030	0.235
69	Vegetables	Leaf vegetables	0.380	0.762	0.483	1.003	1.214	0.051	20.831	0.053	0.028	0.234
70	Vegetables	Mushrooms	0.288	1.874	0.721	1.215	11.061	0.315	2.427	0.006	0.071	0.770
71	Vegetables	Onion-family vegetables	0.228	0.520	0.410	1.035	0.531	0.059	3.625	0.009	0.022	0.110
72	Vegetables	Root vegetables and tubers	0.375	0.484	0.362	1.046	3.924	0.052	19.944	0.051	0.039	0.223
73	water & drinks	Beer, cider and long drink	0.045	0.155	0.005	0.855	0.046	0.015	4.076	0.010	0.000	0.000
74	water & drinks	Coffee beverages	0.096	0.046	0.025	1.065	0.080	0.002	0.657	0.002	0.022	0.114
75	water & drinks	Juice	0.163	0.396	0.086	0.976	0.272	0.035	10.089	0.026	0.535	1.333
76	water & drinks	Soft drinks (incl. sport drinks)	0.043	0.041	0.011	0.638	0.079	0.007	5.425	0.014	0.002	0.056
77	water & drinks	Tea	0.012	0.028	0.009	1.210	0.075	0.005	0.248	0.001	0.000	0.000
78	water & drinks	Water	0.000	0.000	0.005	1.000	0.050	0.000	1.090	0.003	0.000	0.000
79	water & drinks	Wines and spirits	0.074	0.495	0.054	1.879	0.097	0.016	4.552	0.012	0.000	0.051

Female			Nutrient composition (per 100g)								
#	Food group	Food category	Carbohydrates (g)	Dietary fiber (g)	Proteins (g)	Alcohol (g)	Energy (kJ)	Cis-MUFA (g)	Cis-PUFA (g)	Alpha-linolenic acid (ALA) (g)	Linoleic acid + ALA (g)
	group	category	CHOAVL	FIBT	PROT	ALC	ENERC	famcira	fapura	ALA	ALALA
1	Dairy	Cheese, ripened or processed <=17 %	2.789	0.000	18.447	0.000	755.519	2.354	0.255	0.049	0.189
2	Dairy	Cheese, ripened or processed > 17 %	0.037	0.000	24.697	0.000	1532.411	7.079	0.783	0.141	0.562
3	Dairy	Cheese, unripened, fresh cheese <=15 %	2.500	0.000	15.810	0.000	385.270	0.479	0.056	0.012	0.038
4	Dairy	Cheese, unripened, fresh cheese >15 %	3.041	0.098	11.523	0.000	580.223	2.214	0.257	0.089	0.220
5	Dairy	Cream	3.749	0.011	1.872	0.021	916.250	5.651	0.751	0.123	0.493
6	Dairy	Dairy dessert	15.889	0.053	3.987	0.000	532.788	1.290	0.279	0.045	0.231
7	Dairy	Dairy imitates	5.103	0.907	4.086	0.000	338.642	1.775	1.251	0.143	0.671
8	Dairy	Fermented milk products, other	12.072	1.657	3.599	0.000	351.222	0.184	0.836	0.211	0.836
9	Dairy	Milk powder	27.965	0.000	56.712	0.000	1584.368	1.204	0.128	0.006	0.026
10	Dairy	Milk, fat <=2%	4.125	0.000	3.373	0.000	183.245	0.343	0.032	0.005	0.025
11	Dairy	Milk, fat >2%	5.009	0.002	3.010	0.000	269.294	0.754	0.077	0.012	0.056
12	Dairy	Quark	3.100	0.000	9.810	0.000	244.710	0.054	0.014	0.001	0.007
13	Dairy	Skimmed milk	4.250	0.000	3.698	0.000	141.394	0.021	0.004	0.000	0.003
14	Dairy	Soured and cultured milks	5.811	0.185	3.034	0.000	182.197	0.246	0.151	0.029	0.150
15	Dairy	Yoghurt	8.260	0.081	3.124	0.000	247.894	0.269	0.032	0.006	0.025
16	Eggs	Eggs	0.307	0.000	12.710	0.000	642.901	4.618	1.878	0.220	1.593
17	Fats	Blended fat and oil	0.318	0.000	0.371	0.000	2816.445	37.322	19.827	6.267	19.605
18	Fats	Butter, milk fat	0.778	0.000	1.175	0.000	3059.915	19.505	2.585	0.362	1.408
19	Fats	Margarine and similar products	0.300	0.000	0.380	0.000	2971.480	28.046	12.491	2.368	12.491
20	Fats	Salad dressings and mayonnaises	4.084	0.065	1.796	0.000	2094.948	24.912	13.402	3.637	13.235
21	Fats	Vegetable fat and oil	0.000	0.000	0.000	0.000	3699.973	61.042	21.683	5.226	21.378
22	Fish/Seafood	Crustaceans and molluscs	0.000	0.000	15.092	0.000	289.028	0.173	0.322	0.008	0.023
23	Fish/Seafood	Fish	0.000	0.000	16.890	0.000	624.344	3.115	3.168	0.160	0.506
24	Fish/Seafood	Fish products	2.318	0.107	19.719	0.000	724.553	3.948	3.018	0.467	2.042
25	Fruits	Berries	10.328	2.832	0.756	0.000	254.475	0.041	0.154	0.073	0.153
26	Fruits	Canned or dried fruit	18.045	2.075	0.904	0.000	757.126	7.449	1.273	0.092	1.270
27	Fruits	Citrus fruits	8.599	2.263	0.564	0.000	188.179	0.028	0.058	0.027	0.058
28	Fruits	Miscellaneous fruits	13.942	2.038	1.321	0.000	385.847	1.556	0.261	0.052	0.260
29	Fruits	Pome fruits	8.267	1.940	0.207	0.000	171.163	0.006	0.049	0.010	0.048
30	Grains	Breakfast cereal	11.025	1.519	2.294	0.000	283.936	0.359	0.421	0.092	0.421
31	Grains	Fine bakerly wares	41.144	1.637	6.809	0.000	1276.192	3.831	1.583	0.291	1.507
32	Grains	Multigrain bread and rolls	38.890	7.686	8.088	0.000	984.204	0.960	1.364	0.322	1.362
33	Grains	Noodles	26.691	1.238	3.530	0.000	559.502	0.251	0.244	0.014	0.244
34	Grains	Other cereal products	40.019	6.843	12.766	0.000	2060.395	12.735	8.886	0.812	8.882
35	Grains	Other grain and grain flour	48.486	7.857	10.296	0.000	1216.214	1.232	1.640	0.089	1.634
36	Grains	Pasta	20.300	1.379	3.132	0.000	432.132	0.151	0.234	0.012	0.233
37	Grains	Rice and rice flour	25.649	1.682	2.736	0.000	524.621	0.256	0.262	0.012	0.262
38	Grains	Rye and rye flour	57.780	11.957	10.195	0.000	1343.273	0.282	0.972	0.123	0.972
39	Grains	Rye bread and rolls	45.037	10.264	7.629	0.000	1039.632	0.200	0.666	0.080	0.660
40	Grains	Starch	83.116	0.999	0.501	0.000	1453.000	0.113	0.029	0.002	0.029
41	Grains	Wheat and wheat flour	73.065	3.864	11.656	0.000	1531.472	0.170	0.702	0.041	0.702
42	Grains	Wheat bread and rolls	49.766	3.258	8.660	0.000	1131.710	0.884	0.894	0.119	0.892
43	Legumes/Nuts	Legume products	10.741	8.312	18.314	0.000	1530.761	10.189	8.085	0.073	8.085
44	Legumes/Nuts	Legumes	9.579	3.331	5.580	0.000	322.725	0.099	0.552	0.117	0.549
45	Legumes/Nuts	Nuts and seeds	9.879	9.381	21.888	0.000	2438.740	19.820	18.576	4.578	18.531
46	Legumes/Nuts	Soya and soya products	13.290	10.794	32.458	0.000	1093.823	2.430	2.955	0.488	2.902
47	Meats	Beef	0.000	0.000	29.973	0.000	730.258	2.275	0.298	0.039	0.204
48	Meats	Edible offal	3.046	0.326	16.310	0.000	832.530	5.241	1.988	0.129	1.538
49	Meats	Game	0.000	0.000	27.053	0.000	633.091	1.284	0.394	0.058	0.266
50	Meats	Lamb	0.000	0.000	22.740	0.000	938.280	5.587	0.712	0.134	0.432
51	Meats	Meat cuts	0.719	0.000	17.520	0.000	557.188	2.995	0.903	0.095	0.805
52	Meats	Pork	0.000	0.000	26.670	0.000	1154.040	8.487	2.605	0.188	2.253
53	Meats	Poultry	0.000	0.000	23.602	0.000	913.853	7.025	2.733	0.163	2.640
54	Meats	Sausage	4.426	0.073	12.041	0.000	982.380	7.731	2.128	0.168	1.925
55	Meats	Mycoprotein	3.000	6.250	11.250	0.000	355.640	1.400	1.541	4.900	9.200
56	Spices/Condiments	Condiments	6.293	0.333	1.585	0.000	568.827	4.596	2.444	0.628	2.399
57	Spices/Condiments	Dried spices and herbs	2.145	3.005	3.299	0.000	159.201	0.110	0.258	0.171	0.257
58	Spices/Condiments	Miscellaneous ingredients	8.257	3.955	12.134	0.000	747.338	3.625	0.869	0.019	0.868
59	Spices/Condiments	Salt	0.000	0.000	0.570	0.000	9.697	0.000	0.000	0.000	0.000
60	Starchy/Tubers	Potato products	41.155	4.051	4.626	0.000	1837.144	9.973	9.510	1.639	9.505
61	Starchy/Tubers	Potatoes	15.500	1.400	1.879	0.000	315.337	0.002	0.064	0.029	0.064
62	Sugar	Chocolate	73.871	2.459	2.028	0.000	1897.660	5.473	1.214	0.139	1.211
63	Sugar	Jams	37.292	1.353	0.534	0.000	662.247	0.036	0.063	0.026	0.063
64	Sugar	Non-chocolate confectionery	81.018	0.919	1.904	0.000	1501.006	0.692	0.233	0.006	0.233
65	Sugar	Sugar and syrups	93.983	0.000	0.139	0.000	1600.081	0.000	0.000	0.000	0.000
66	Vegetables	Cabbages	4.614	1.920	1.215	0.000	124.128	0.008	0.078	0.050	0.075
67	Vegetables	Canned or other vegetable products	7.945	1.003	1.095	0.000	207.016	0.272	0.135	0.016	0.135
68	Vegetables	Fruit vegetables	3.447	0.861	0.704	0.000	93.819	0.021	0.078	0.015	0.078
69	Vegetables	Leaf vegetables	1.036	1.248	1.255	0.000	60.276	0.008	0.112	0.074	0.108
70	Vegetables	Mushrooms	2.542	1.974	2.648	0.000	139.686	0.015	0.160	0.000	0.153
71	Vegetables	Onion-family vegetables	5.034	1.200	1.386	0.000	129.614	0.016	0.053	0.004	0.053
72	Vegetables	Root vegetables and tubers	5.168	2.379	1.252	0.000	142.471	0.012	0.128	0.036	0.126
73	water & drinks	Beer, cider and long drink	3.952	0.001	0.304	3.573	178.762	0.000	0.000	0.000	0.000
74	water & drinks	Coffee beverages	0.398	0.013	0.326	0.000	16.675	0.008	0.001	0.000	0.001
75	water & drinks	Juice	9.356	0.314	0.755	0.000	230.658	0.470	0.201	0.027	0.201
76	water & drinks	Soft drinks (incl. sport drinks)	5.632	0.007	0.185	0.000	101.402	0.003	0.006	0.002	0.006
77	water & drinks	Tea	4.354	0.000	0.050	0.000	75.178	0.000	0.000	0.000	0.000
78	water & drinks	Water	0.030	0.000	0.000	0.000	0.503	0.000	0.000	0.000	0.000
79	water & drinks	Wines and spirits	0.878	0.000	0.302	9.655	310.410	0.000	0.000	0.000	0.000

Female			Nutrient composition (per 100g)							
#	Food group	Food category	Docosahexaenoic acid (mg)	Histidine (mg)	Isoleucine (mg)	Leucine (mg)	Lysine (mg)	Methionine + cysteine (mg)	Phenylalanine + tyrosine (mg)	Threonine (mg)
	group	category	DHA	HIS	ILE	LEU	LYS	met_cys	tyr_phe	THR
1	Dairy	Cheese, ripened or processed <=17 %	0.000	708.293	1007.823	1845.980	1848.409	640.694	2067.327	749.006
2	Dairy	Cheese, ripened or processed > 17 %	0.000	727.457	1112.490	2071.259	1826.768	746.766	2278.250	855.142
3	Dairy	Cheese, unripened, fresh cheese <=15 %	0.000	326.000	591.000	1116.000	934.000	335.000	1181.000	500.000
4	Dairy	Cheese, unripened, fresh cheese >15 %	0.452	211.138	402.460	723.860	524.801	248.533	756.664	337.925
5	Dairy	Cream	3.454	109.937	198.755	368.459	310.677	122.551	361.965	159.954
6	Dairy	Dairy dessert	5.517	102.869	224.357	375.754	305.869	134.450	365.338	171.199
7	Dairy	Dairy imitates	0.000	60.629	113.253	181.872	135.760	44.072	202.698	94.645
8	Dairy	Fermented milk products, other	0.000	102.822	181.817	333.317	295.248	116.030	358.035	148.054
9	Dairy	Milk powder	0.000	517.309	1264.736	2060.567	1708.968	767.462	1798.592	1107.227
10	Dairy	Milk, fat <=2%	0.000	100.000	171.000	313.000	276.000	107.000	338.000	141.000
11	Dairy	Milk, fat >2%	0.000	95.479	163.767	300.342	265.150	102.479	323.534	134.671
12	Dairy	Quark	0.000	326.000	591.000	1116.000	934.000	335.000	1181.000	500.000
13	Dairy	Skimmed milk	0.000	102.000	174.000	319.000	282.000	109.000	345.000	144.000
14	Dairy	Soured and cultured milks	0.000	125.380	229.022	420.433	372.443	148.331	449.363	184.431
15	Dairy	Yoghurt	0.000	165.403	330.566	609.726	532.697	218.322	621.331	263.048
16	Eggs	Eggs	108.058	285.214	632.391	1011.577	844.288	615.153	1103.254	536.328
17	Fats	Blended fat and oil	0.000	23.000	52.000	84.000	68.000	29.000	82.000	39.000
18	Fats	Butter, milk fat	0.000	22.596	50.083	81.490	65.787	28.488	80.544	37.326
19	Fats	Margarine and similar products	0.000	23.000	52.000	84.000	68.000	29.000	82.000	39.000
20	Fats	Salad dressings and mayonnaises	16.142	16.218	35.112	51.526	42.112	29.199	59.526	32.720
21	Fats	Vegetable fat and oil	0.000	0.000	0.000	0.000	0.000	0.000	0.000	0.000
22	Fish/Seafood	Crustaceans and molluscs	108.587	301.455	710.728	1161.455	1267.092	578.728	1107.546	611.908
23	Fish/Seafood	Fish	1393.934	564.029	949.715	1623.958	1861.599	831.264	1541.742	870.954
24	Fish/Seafood	Fish products	438.175	743.885	1179.992	2091.924	2337.890	1132.846	1879.276	1118.921
25	Fruits	Berries	0.000	11.733	20.395	38.087	20.603	15.488	46.192	20.094
26	Fruits	Canned or dried fruit	0.000	9.820	12.405	22.727	23.770	10.472	24.463	14.355
27	Fruits	Citrus fruits	0.000	12.321	17.962	19.419	33.562	17.742	36.176	12.339
28	Fruits	Miscellaneous fruits	0.000	59.727	34.209	69.901	57.240	25.408	65.431	32.543
29	Fruits	Pome fruits	0.000	4.581	6.699	14.699	13.559	2.280	8.699	6.699
30	Grains	Breakfast cereal	0.000	195.749	341.466	546.774	280.181	305.844	639.305	320.890
31	Grains	Fine bakerly wares	12.047	137.371	260.627	455.162	260.680	239.767	495.564	209.570
32	Grains	Multigrain bread and rolls	0.000	206.366	336.779	579.621	301.303	327.287	649.574	282.318
33	Grains	Noodles	0.012	74.470	136.657	245.146	83.311	148.323	272.108	100.110
34	Grains	Other cereal products	0.085	341.955	536.895	1054.857	472.578	438.195	1153.992	441.663
35	Grains	Other grain and grain flour	5.264	348.931	545.118	1131.540	660.844	572.806	1211.020	456.189
36	Grains	Pasta	0.120	282.663	471.632	956.085	282.673	382.930	863.922	429.491
37	Grains	Rice and rice flour	0.000	63.042	116.063	222.130	97.054	118.065	234.195	96.056
38	Grains	Rye and rye flour	0.000	254.750	378.633	852.907	397.225	455.857	898.406	489.582
39	Grains	Rye bread and rolls	0.000	183.975	322.511	585.363	235.523	315.401	630.802	257.853
40	Grains	Starch	0.000	165.354	296.717	426.161	410.291	175.899	537.284	277.171
41	Grains	Wheat and wheat flour	0.000	233.447	360.798	714.425	240.071	404.111	832.865	287.330
42	Grains	Wheat bread and rolls	0.000	191.722	308.815	564.706	248.971	314.657	630.999	262.558
43	Legumes/Nuts	Legume products	0.000	347.400	557.393	974.285	698.674	422.341	1069.383	516.894
44	Legumes/Nuts	Legumes	0.000	169.116	279.751	477.641	443.008	164.868	489.780	267.142
45	Legumes/Nuts	Nuts and seeds	0.000	521.765	837.244	1465.284	822.433	666.013	1674.891	733.777
46	Legumes/Nuts	Soya and soya products	0.000	455.765	867.519	1415.555	913.194	348.929	1548.041	788.795
47	Meats	Beef	0.000	871.138	1016.229	1840.009	1931.872	1106.000	1768.370	889.147
48	Meats	Edible offal	60.758	561.572	724.333	1332.746	1332.772	459.654	1193.131	718.718
49	Meats	Game	0.000	565.000	1371.000	2258.000	2016.000	1049.000	1774.000	1290.000
50	Meats	Lamb	0.000	841.000	1339.000	2295.000	2452.000	1106.000	2201.000	1314.000
51	Meats	Meat cuts	4.540	686.211	820.680	1470.152	1572.062	678.879	1341.530	820.023
52	Meats	Pork	17.048	1026.000	1203.000	2061.000	2310.000	1008.000	1920.000	1173.000
53	Meats	Poultry	21.540	633.377	1074.141	1590.148	1768.450	880.553	1552.282	907.267
54	Meats	Sausage	4.272	427.331	602.715	1052.276	1159.610	495.004	972.126	587.705
55	Meats	Mycoprotein	0.000	390.000	570.000	950.000	910.000	230.000	540.000	610.000
56	Spices/Condiments	Condiments	0.772	34.672	60.116	97.158	85.617	51.743	101.972	62.430
57	Spices/Condiments	Dried spices and herbs	0.000	67.125	146.232	229.578	182.341	66.467	241.601	116.285
58	Spices/Condiments	Miscellaneous ingredients	0.000	128.194	274.209	389.027	395.683	167.806	433.506	255.062
59	Spices/Condiments	Salt	0.000	0.000	0.000	0.000	0.000	0.000	0.000	0.000
60	Starchy/Tubers	Potato products	0.000	211.445	306.994	756.867	297.724	283.164	711.015	294.397
61	Starchy/Tubers	Potatoes	0.000	35.000	66.000	98.000	107.000	56.000	129.000	67.000
62	Sugar	Chocolate	0.000	67.000	151.000	236.000	195.000	88.000	333.000	154.000
63	Sugar	Jams	0.000	11.450	16.523	28.133	27.210	10.271	39.206	18.084
64	Sugar	Non-chocolate confectionery	0.000	195.212	221.889	545.300	250.595	177.315	702.675	190.645
65	Sugar	Sugar and syrups	0.000	22.066	44.807	75.471	58.544	47.597	922.652	38.183
66	Vegetables	Cabbages	0.000	22.009	33.760	43.970	46.783	22.874	52.015	35.580
67	Vegetables	Canned or other vegetable products	0.000	18.903	25.421	36.154	37.118	14.565	43.359	28.788
68	Vegetables	Fruit vegetables	0.000	16.441	28.221	45.416	42.732	20.681	54.350	29.088
69	Vegetables	Leaf vegetables	0.000	22.184	55.067	63.304	60.470	24.728	70.457	47.060
70	Vegetables	Mushrooms	0.000	53.022	74.699	119.561	102.755	41.619	127.095	102.755
71	Vegetables	Onion-family vegetables	0.000	16.019	18.139	30.771	43.771	8.753	43.588	23.773
72	Vegetables	Root vegetables and tubers	0.000	40.163	69.722	98.247	107.513	76.816	104.992	136.948
73	water & drinks	Beer, cider and long drink	0.000	0.000	0.000	0.000	0.000	0.000	0.000	0.000
74	water & drinks	Coffee beverages	0.000	3.212	5.076	10.463	4.945	5.065	10.398	2.853
75	water & drinks	Juice	0.000	2.928	6.157	12.188	8.822	7.152	11.532	7.187
76	water & drinks	Soft drinks (incl. sport drinks)	0.000	0.630	1.575	3.150	2.205	1.890	2.835	1.890
77	water & drinks	Tea	0.000	2.000	2.000	5.000	1.000	2.000	5.000	1.000
78	water & drinks	Water	0.000	0.000	0.000	0.000	0.000	0.000	0.000	0.000
79	water & drinks	Wines and spirits	0.000	0.871	1.948	3.151	2.555	1.100	3.116	1.455

Supplemental Data 1: data for different sustainability metrics of foods by category (female)

Female			Nutrient composition (per 100g)		
#	Food group	Food category	Tryptophan (mg)	Valine (mg)	Total essential amino acids (mg)
	group	category	TRP	VAL	ess_aa_sum
1	Dairy	Cheese, ripened or processed <=17 %	287.106	1324.352	9293.689
2	Dairy	Cheese, ripened or processed > 17 %	347.227	1446.139	10085.726
3	Dairy	Cheese, unripened, fresh cheese <=15 %	147.000	748.000	5208.000
4	Dairy	Cheese, unripened, fresh cheese >15 %	143.248	480.289	3395.031
5	Dairy	Cream	50.473	231.821	1706.434
6	Dairy	Dairy dessert	52.645	253.864	1769.643
7	Dairy	Dairy imitates	33.570	112.278	877.742
8	Dairy	Fermented milk products, other	40.871	237.411	1614.752
9	Dairy	Milk powder	316.691	1341.789	9720.306
10	Dairy	Milk, fat <=2%	42.000	216.000	1517.000
11	Dairy	Milk, fat >2%	40.192	206.959	1453.710
12	Dairy	Quark	147.000	748.000	5208.000
13	Dairy	Skimmed milk	43.000	221.000	1548.000
14	Dairy	Soured and cultured milks	46.940	306.183	2031.893
15	Dairy	Yoghurt	67.507	442.781	2900.118
16	Eggs	Eggs	153.402	769.672	5220.464
17	Fats	Blended fat and oil	12.000	57.000	397.000
18	Fats	Butter, milk fat	11.784	55.976	385.937
19	Fats	Margarine and similar products	12.000	57.000	397.000
20	Fats	Salad dressings and mayonnaises	9.392	39.221	278.089
21	Fats	Vegetable fat and oil	0.000	0.000	0.000
22	Fish/Seafood	Crustaceans and molluscs	201.000	693.091	5965.367
23	Fish/Seafood	Fish	217.125	1072.674	8592.362
24	Fish/Seafood	Fish products	287.431	1321.179	10855.680
25	Fruits	Berries	5.040	28.159	184.288
26	Fruits	Canned or dried fruit	5.061	18.079	128.490
27	Fruits	Citrus fruits	6.020	27.242	164.283
28	Fruits	Miscellaneous fruits	11.361	50.543	377.662
29	Fruits	Pome fruits	1.140	12.699	68.773
30	Grains	Breakfast cereal	96.305	420.866	2679.064
31	Grains	Fine bakery wares	76.770	298.204	2116.208
32	Grains	Multigrain bread and rolls	129.413	414.224	2797.661
33	Grains	Noodles	45.024	159.390	1071.881
34	Grains	Other cereal products	175.452	667.167	4582.794
35	Grains	Other grain and grain flour	228.313	732.634	5129.478
36	Grains	Pasta	165.167	550.510	3901.836
37	Grains	Rice and rice flour	31.020	164.090	996.580
38	Grains	Rye and rye flour	187.341	533.790	3912.627
39	Grains	Rye bread and rolls	96.987	383.169	2621.306
40	Grains	Starch	113.706	353.460	2464.352
41	Grains	Wheat and wheat flour	129.640	422.822	3092.691
42	Grains	Wheat bread and rolls	117.361	370.746	2594.742
43	Legumes/Nuts	Legume products	195.713	641.416	4832.213
44	Legumes/Nuts	Legumes	62.602	322.345	2439.407
45	Legumes/Nuts	Nuts and seeds	264.840	1041.709	7078.907
46	Legumes/Nuts	Soya and soya products	207.645	901.564	6613.707
47	Meats	Beef	256.224	1079.313	9627.492
48	Meats	Edible offal	177.478	928.131	6891.727
49	Meats	Game	403.000	1452.000	11022.000
50	Meats	Lamb	334.000	1440.000	11988.000
51	Meats	Meat cuts	189.046	890.307	7650.539
52	Meats	Pork	326.000	1394.000	11198.000
53	Meats	Poultry	241.453	1066.830	8713.840
54	Meats	Sausage	140.650	654.295	5496.062
55	Meats	Mycoprotein	180.000	600.000	4980.000
56	Spices/Condiments	Condiments	20.947	73.998	519.101
57	Spices/Condiments	Dried spices and herbs	38.329	175.284	1131.418
58	Spices/Condiments	Miscellaneous ingredients	64.619	299.515	2138.928
59	Spices/Condiments	Salt	0.000	0.000	0.000
60	Starchy/Tubers	Potato products	78.620	434.246	2955.138
61	Starchy/Tubers	Potatoes	21.000	103.000	610.000
62	Sugar	Chocolate	58.000	234.000	1322.000
63	Sugar	Jams	6.986	23.423	156.891
64	Sugar	Non-chocolate confectionery	80.605	281.919	2279.681
65	Sugar	Sugar and syrups	15.214	59.549	1228.598
66	Vegetables	Cabbages	11.145	43.338	280.706
67	Vegetables	Canned or other vegetable products	8.673	27.412	216.137
68	Vegetables	Fruit vegetables	7.851	32.917	247.857
69	Vegetables	Leaf vegetables	11.892	54.218	372.844
70	Vegetables	Mushrooms	29.804	206.542	800.550
71	Vegetables	Onion-family vegetables	15.060	26.505	205.770
72	Vegetables	Root vegetables and tubers	15.461	78.723	631.570
73	water & drinks	Beer, cider and long drink	0.000	0.000	0.000
74	water & drinks	Coffee beverages	0.725	6.743	41.171
75	water & drinks	Juice	1.258	9.644	54.434
76	water & drinks	Soft drinks (incl. sport drinks)	0.315	2.520	14.804
77	water & drinks	Tea	0.000	3.000	17.000
78	water & drinks	Water	0.000	0.000	0.000
79	water & drinks	Wines and spirits	0.458	2.154	14.951

Male			Dietary intake (g/cap/day)						Nutrient composition (per 100g)		
#	Food group	Food category	Mean	5th percentile	95th percentile	Standard Deviation	Price (€/kg)	Global Warming Potential (kg CO2eq/kg)	Vitamin A (µg)	Vitamin D (µg)	Vitamin E (mg)
	group	category	q0	P5	P95	sd	price	lca	VITA	VITD	VITE
1	Dairy	Cheese, ripened or processed <=17 %	5.518	0.000	28.886	11.955	9.056	6.664	95.620	0.097	0.212
2	Dairy	Cheese, ripened or processed > 17 %	31.362	0.000	180.241	34.294	9.016	6.664	226.731	0.227	0.548
3	Dairy	Cheese, unripened, fresh cheese <=15 %	6.928	0.000	42.500	31.588	8.466	5.816	20.840	0.016	0.070
4	Dairy	Cheese, unripened, fresh cheese >15 %	4.207	0.000	7.536	19.152	9.013	5.801	86.482	0.057	0.168
5	Dairy	Cream	14.759	0.000	51.043	23.603	5.080	7.476	191.905	0.302	0.588
6	Dairy	Dairy dessert	18.004	0.000	53.127	45.960	5.084	2.924	33.293	0.418	0.280
7	Dairy	Dairy imitates	10.840	0.000	6.395	51.639	2.122	1.897	0.157	0.922	0.395
8	Dairy	Fermented milk products, other	1.169	0.000	1.169	13.907	4.310	3.769	36.244	0.304	0.161
9	Dairy	Milk powder	1.187	0.000	1.187	5.823	10.520	15.791	5.746	0.800	0.010
10	Dairy	Milk, fat <=2%	152.885	0.000	625.000	222.951	1.009	1.564	14.356	1.009	0.030
11	Dairy	Milk, fat >2%	19.042	0.000	76.703	82.011	0.836	1.564	29.482	0.870	0.068
12	Dairy	Quark	18.124	0.000	125.000	62.476	8.466	3.784	1.480	0.020	0.010
13	Dairy	Skimmed milk	94.570	0.000	546.285	203.182	1.011	1.564	0.787	1.063	0.001
14	Dairy	Soured and cultured milks	27.249	0.000	270.000	78.963	1.842	1.790	2.416	0.025	0.087
15	Dairy	Yoghurt	55.792	0.000	347.037	98.495	2.328	2.871	11.264	0.408	0.068
16	Eggs	Eggs	21.287	0.000	144.048	35.792	3.837	2.575	257.116	2.347	2.310
17	Fats	Blended fat and oil	20.607	0.000	89.232	22.904	5.722	5.438	180.397	17.414	12.585
18	Fats	Butter, milk fat	2.845	0.000	13.417	8.502	4.930	15.594	706.146	0.325	1.511
19	Fats	Margarine and similar products	10.115	0.000	57.386	18.167	4.210	1.645	900.000	7.500	8.320
20	Fats	Salad dressings and mayonnaises	6.597	0.000	36.650	11.028	5.000	2.550	170.100	0.543	9.743
21	Fats	Vegetable fat and oil	2.598	0.000	15.066	5.111	5.358	2.146	0.804	0.000	16.408
22	Fish/Seafood	Crustaceans and molluscs	1.079	0.000	1.079	6.233	18.449	7.382	2.337	0.050	3.950
23	Fish/Seafood	Fish	20.505	0.000	69.261	42.016	10.514	6.582	9.246	7.584	1.573
24	Fish/Seafood	Fish products	11.881	0.000	37.894	30.252	15.130	7.323	16.087	7.148	1.506
25	Fruits	Berries	22.039	0.000	118.899	41.186	4.107	2.510	3.506	0.000	1.285
26	Fruits	Canned or dried fruit	8.321	0.000	45.477	22.199	5.681	2.670	6.327	0.000	2.211
27	Fruits	Citrus fruits	23.193	0.000	154.000	56.550	2.437	1.113	11.293	0.000	0.347
28	Fruits	Miscellaneous fruits	42.382	0.000	175.939	67.155	2.791	2.145	3.972	0.000	0.347
29	Fruits	Pome fruits	27.597	0.000	132.675	66.384	1.950	0.409	1.448	0.000	0.216
30	Grains	Breakfast cereal	90.780	0.000	465.846	137.193	0.218	0.154	1.604	0.118	0.199
31	Grains	Fine bakerly wares	49.293	0.000	266.215	57.191	8.233	1.533	89.487	0.761	1.235
32	Grains	Multigrain bread and rolls	42.781	0.000	217.253	54.719	4.881	0.614	1.380	0.000	0.940
33	Grains	Noodles	7.842	0.000	7.842	39.059	1.838	0.321	0.161	0.000	0.190
34	Grains	Other cereal products	4.509	0.000	13.588	11.983	6.816	1.704	3.720	0.002	2.648
35	Grains	Other grain and grain flour	0.887	0.000	0.887	5.559	3.684	1.520	0.165	0.000	0.639
36	Grains	Pasta	33.011	0.000	204.568	69.137	1.838	0.596	0.288	0.002	0.150
37	Grains	Rice and rice flour	60.631	0.000	391.131	127.462	1.162	2.283	0.000	0.000	0.134
38	Grains	Rye and rye flour	0.161	0.000	0.161	0.928	1.000	1.051	0.516	0.000	0.874
39	Grains	Rye bread and rolls	43.273	0.000	152.500	54.096	4.240	0.704	0.316	0.000	0.701
40	Grains	Starch	1.377	0.000	7.953	3.260	2.860	1.704	0.348	0.000	0.032
41	Grains	Wheat and wheat flour	12.473	0.000	57.737	22.415	0.761	0.596	0.360	0.000	0.439
42	Grains	Wheat bread and rolls	22.766	0.000	136.872	35.603	4.300	0.415	1.402	0.015	0.664
43	Legumes/Nuts	Legume products	0.298	0.000	0.298	3.000	3.554	1.028	0.425	0.000	5.143
44	Legumes/Nuts	Legumes	17.260	0.000	86.441	37.050	3.275	0.458	28.537	0.000	0.056
45	Legumes/Nuts	Nuts and seeds	4.080	0.000	7.606	13.077	13.423	2.511	0.961	0.000	7.713
46	Legumes/Nuts	Soya and soya products	2.219	0.000	2.219	12.697	2.473	3.998	4.322	0.000	0.995
47	Meats	Beef	21.744	0.000	119.332	32.383	14.552	58.974	12.192	0.283	0.574
48	Meats	Edible offal	2.833	0.000	2.833	12.490	7.492	27.205	7855.622	0.433	0.639
49	Meats	Game	2.595	0.000	2.595	14.847	13.463	9.178	7.336	0.254	1.016
50	Meats	Lamb	1.034	0.000	1.034	6.466	15.668	55.688	12.774	0.239	0.382
51	Meats	Meat cuts	27.987	0.000	128.120	32.241	9.424	8.121	4.512	0.319	0.392
52	Meats	Pork	15.128	0.000	70.540	28.395	12.123	18.771	12.011	0.579	0.528
53	Meats	Poultry	30.127	0.000	111.598	45.563	6.153	6.311	36.067	0.803	0.814
54	Meats	Sausage	34.728	0.000	195.364	49.385	7.108	10.768	8.037	0.224	0.354
55	Meats	Mycoprotein	0.000	0.000	495.241	37.594	13.149	2.291	0.000	0.000	0.000
56	Spices/Condiments	Condiments	22.896	0.000	139.178	44.791	6.186	1.411	56.030	0.251	1.398
57	Spices/Condiments	Dried spices and herbs	0.384	0.000	0.793	1.493	25.144	1.411	368.721	0.000	2.389
58	Spices/Condiments	Miscellaneous ingredients	0.895	0.000	6.540	1.701	8.081	0.000	0.000	0.000	0.351
59	Spices/Condiments	Salt	1.509	0.000	4.032	1.059	2.180	0.000	0.000	0.000	0.000
60	Starchy/Tubers	Potato products	2.985	0.000	10.656	12.798	3.847	1.733	3.852	0.001	4.106
61	Starchy/Tubers	Potatoes	82.613	0.000	222.671	81.789	0.830	1.122	0.640	0.000	0.050
62	Sugar	Chocolate	6.717	0.000	36.885	15.772	15.080	17.469	4.827	0.002	0.658
63	Sugar	Jams	4.354	0.000	14.000	13.555	7.119	2.067	5.475	0.000	0.370
64	Sugar	Non-chocolate confectionery	7.568	0.000	37.169	18.683	13.832	3.234	0.047	0.000	0.035
65	Sugar	Sugar and syrups	6.163	0.000	29.693	10.062	1.365	0.803	0.000	0.000	0.000
66	Vegetables	Cabbages	6.279	0.000	32.930	19.759	2.310	1.297	5.007	0.000	0.057
67	Vegetables	Canned or other vegetable products	23.061	0.000	117.998	30.747	4.180	2.278	179.082	0.028	0.923
68	Vegetables	Fruit vegetables	83.696	0.000	284.653	69.689	2.608	2.066	42.206	0.000	0.477
69	Vegetables	Leaf vegetables	17.511	0.000	64.746	22.148	7.322	1.195	52.175	0.000	0.686
70	Vegetables	Mushrooms	1.963	0.000	7.107	8.254	4.569	0.121	21.047	3.877	0.000
71	Vegetables	Onion-family vegetables	11.464	0.000	35.365	13.082	1.573	0.542	0.567	0.000	0.041
72	Vegetables	Root vegetables and tubers	36.542	0.000	160.764	46.038	1.806	0.506	463.349	0.000	0.367
73	water & drinks	Beer, cider and long drink	138.922	0.000	500.000	294.360	4.769	1.269	0.008	0.000	0.002
74	water & drinks	Coffee beverages	530.623	0.000	1344.577	379.845	0.454	0.603	0.265	0.008	0.001
75	water & drinks	Juice	115.054	0.000	500.056	184.692	1.565	0.936	4.793	0.026	0.274
76	water & drinks	Soft drinks (incl. sport drinks)	99.779	0.000	589.671	199.183	1.765	0.711	0.179	0.000	0.020
77	water & drinks	Tea	86.131	0.000	564.867	186.557	0.083	0.102	0.007	0.000	0.001
78	water & drinks	Water	1047.346	1047.346	1047.346	778.516	0.000	0.000	0.000	0.000	0.013
79	water & drinks	Wines and spirits	16.199	0.000	61.269	51.920	11.923	1.276	1.557	0.000	0.004

Supplemental Data 2: data for different sustainability metrics of foods by category (male)

Male			Nutrient composition (per 100g)									
#	Food group	Food category	Thiamin/ B1 (mg)	Riboflavin/ B2 (mg)	Niacin/B3 (mg)	Vitamin B6 (mg)	Folate (µg)	Vitamin B12 (µg)	Vitamin C (mg)	Calcium (mg)	Phosphorus (mg)	Magnesium (mg)
	group	category	THIA	RIBF	NIAEQ	VITPYRID	FOL	VITB12	VITC	CA	P	MG
1	Dairy	Cheese, ripened or processed <=17 %	0.027	0.249	3.228	0.045	18.137	0.647	0.244	544.093	359.111	19.951
2	Dairy	Cheese, ripened or processed > 17 %	0.026	0.330	6.019	0.054	16.883	1.305	0.040	853.286	493.883	30.584
3	Dairy	Cheese, unripened, fresh cheese <=15 %	0.030	0.224	3.804	0.060	27.000	0.700	0.000	70.000	200.000	9.000
4	Dairy	Cheese, unripened, fresh cheese >15 %	0.050	0.190	2.622	0.052	11.434	0.489	0.409	140.119	142.116	11.503
5	Dairy	Cream	0.037	0.142	0.479	0.028	6.409	0.422	0.929	79.482	68.327	8.961
6	Dairy	Dairy dessert	0.041	0.221	1.021	0.090	5.823	0.525	1.206	127.651	50.383	9.369
7	Dairy	Dairy imitates	0.070	0.161	1.160	0.031	28.545	0.359	0.094	128.268	75.827	26.276
8	Dairy	Fermented milk products, other	0.065	0.117	0.854	0.044	19.995	0.197	0.980	111.872	62.516	15.936
9	Dairy	Milk powder	0.340	2.180	9.748	0.470	12.606	2.449	1.483	1359.637	979.866	126.015
10	Dairy	Milk, fat <=2%	0.035	0.187	0.906	0.050	4.240	0.438	1.088	127.500	90.000	11.000
11	Dairy	Milk, fat >2%	0.033	0.184	0.842	0.050	5.931	0.400	0.974	123.613	90.092	11.113
12	Dairy	Quark	0.046	0.226	2.772	0.050	16.000	0.600	1.000	117.000	180.000	12.000
13	Dairy	Skimmed milk	0.034	0.187	0.980	0.050	7.444	0.436	1.225	140.150	89.725	14.250
14	Dairy	Soured and cultured milks	0.034	0.142	0.843	0.041	11.832	0.283	0.709	89.504	86.857	13.199
15	Dairy	Yoghurt	0.034	0.179	0.838	0.051	5.078	0.247	1.926	121.632	89.538	11.840
16	Eggs	Eggs	0.117	0.378	3.180	0.141	58.984	2.306	0.000	58.447	212.990	13.263
17	Fats	Blended fat and oil	0.001	0.013	0.054	0.000	0.715	0.000	0.022	10.998	9.267	1.863
18	Fats	Butter, milk fat	0.000	0.030	0.100	0.000	2.999	0.000	0.000	23.950	33.943	2.595
19	Fats	Margarine and similar products	0.000	0.000	0.073	0.000	2.000	0.000	0.000	11.000	10.000	2.200
20	Fats	Salad dressings and mayonnaises	0.027	0.092	0.450	0.032	11.270	0.466	0.558	45.722	68.826	6.540
21	Fats	Vegetable fat and oil	0.000	0.000	0.000	0.000	0.000	0.000	0.000	0.000	0.000	0.000
22	Fish/Seafood	Crustaceans and molluscs	0.010	0.030	6.034	0.092	21.401	2.956	0.000	58.000	110.000	22.000
23	Fish/Seafood	Fish	0.123	0.099	8.938	0.483	12.814	3.807	0.000	43.231	210.402	23.622
24	Fish/Seafood	Fish products	0.106	0.110	11.678	0.377	8.941	6.145	0.053	33.357	197.347	26.786
25	Fruits	Berries	0.082	0.025	0.548	0.054	13.828	0.000	21.096	19.025	24.119	10.724
26	Fruits	Canned or dried fruit	0.046	0.032	0.419	0.101	2.816	0.000	6.888	15.108	20.277	15.379
27	Fruits	Citrus fruits	0.083	0.037	0.530	0.089	24.791	0.000	47.544	48.337	20.806	12.951
28	Fruits	Miscellaneous fruits	0.040	0.064	0.908	0.438	13.498	0.000	12.915	8.136	24.418	28.543
29	Fruits	Pome fruits	0.015	0.017	0.220	0.055	2.772	0.000	7.734	5.798	9.532	4.833
30	Grains	Breakfast cereal	0.062	0.046	1.068	0.054	7.850	0.052	0.140	23.525	74.709	23.586
31	Grains	Fine bakerly wares	0.051	0.125	1.802	0.073	23.564	0.288	1.604	92.728	132.052	19.106
32	Grains	Multigrain bread and rolls	0.182	0.100	2.288	0.120	32.530	0.000	0.233	25.827	198.111	65.971
33	Grains	Noodles	0.041	0.024	1.088	0.021	4.725	0.000	0.000	12.437	59.414	12.001
34	Grains	Other cereal products	0.257	0.093	3.712	0.265	28.084	0.002	0.282	56.974	254.357	104.079
35	Grains	Other grain and grain flour	0.274	0.092	3.469	0.136	25.435	0.000	0.000	26.990	249.942	86.940
36	Grains	Pasta	0.016	0.010	1.148	0.092	4.614	0.002	0.000	6.366	50.803	18.519
37	Grains	Rice and rice flour	0.052	0.013	1.675	0.134	4.734	0.000	0.000	5.585	87.257	35.058
38	Grains	Rye and rye flour	0.332	0.160	2.835	0.164	37.384	0.000	0.000	28.633	277.283	86.729
39	Grains	Rye bread and rolls	0.178	0.121	1.976	0.121	36.095	0.000	0.056	25.556	204.384	64.802
40	Grains	Starch	0.040	0.040	0.652	0.048	16.685	0.000	0.033	40.526	70.933	8.606
41	Grains	Wheat and wheat flour	0.091	0.053	3.239	0.108	18.797	0.000	0.000	19.407	126.516	32.548
42	Grains	Wheat bread and rolls	0.166	0.083	3.518	0.087	30.215	0.002	0.004	19.698	118.723	32.033
43	Legumes/Nuts	Legume products	0.636	0.145	11.131	0.229	129.295	0.000	0.948	127.723	320.266	157.590
44	Legumes/Nuts	Legumes	0.188	0.044	2.647	0.165	49.855	0.000	14.648	38.788	126.112	41.003
45	Legumes/Nuts	Nuts and seeds	0.773	0.161	11.833	0.349	86.795	0.000	0.411	93.423	427.317	220.687
46	Legumes/Nuts	Soya and soya products	0.398	0.183	8.111	0.362	158.883	0.009	0.673	361.656	393.409	163.948
47	Meats	Beef	0.135	0.228	13.869	0.581	5.297	1.939	0.000	9.722	244.624	29.197
48	Meats	Edible offal	0.311	1.545	11.446	0.590	504.778	33.980	12.165	14.561	297.117	28.707
49	Meats	Game	0.143	0.289	10.130	0.727	6.489	6.410	0.000	13.362	335.399	38.330
50	Meats	Lamb	0.138	0.290	7.928	0.270	2.030	0.950	0.000	11.040	227.300	27.764
51	Meats	Meat cuts	0.150	0.197	9.215	0.470	3.408	0.582	0.000	7.302	176.907	14.866
52	Meats	Pork	1.255	0.353	6.836	0.490	2.250	0.870	0.000	12.410	224.000	26.995
53	Meats	Poultry	0.038	0.140	7.161	0.551	7.411	0.238	0.000	12.772	208.773	19.767
54	Meats	Sausage	0.331	0.122	5.702	0.272	4.005	0.603	0.008	18.812	121.643	17.875
55	Meats	Mycoprotein	0.040	0.900	1.400	0.000	0.000	0.000	0.000	42.500	260.000	6.000
56	Spices/Condiments	Condiments	0.013	0.039	0.559	0.023	3.652	0.044	1.635	28.405	36.522	8.892
57	Spices/Condiments	Dried spices and herbs	0.060	0.277	2.090	0.167	116.042	0.000	120.801	167.498	60.580	36.204
58	Spices/Condiments	Miscellaneous ingredients	0.355	1.507	14.255	0.341	887.833	0.012	0.000	25.907	366.076	56.076
59	Spices/Condiments	Salt	0.000	0.000	0.000	0.000	0.000	0.000	0.000	156.341	70.082	44.804
60	Starchy/Tubers	Potato products	0.178	0.059	4.025	0.509	34.579	0.000	3.366	24.223	130.958	47.921
61	Starchy/Tubers	Potatoes	0.210	0.030	0.917	0.120	23.170	0.000	10.000	5.600	45.000	24.000
62	Sugar	Chocolate	0.006	0.046	0.558	0.024	3.522	0.030	0.102	29.486	72.091	43.338
63	Sugar	Jams	0.020	0.018	0.351	0.037	4.985	0.000	6.439	12.089	14.240	8.662
64	Sugar	Non-chocolate confectionery	0.001	0.010	0.261	0.003	0.813	0.005	0.012	149.216	22.753	51.622
65	Sugar	Sugar and syrups	0.002	0.007	0.032	0.000	0.000	0.000	0.639	5.030	1.301	2.359
66	Vegetables	Cabbages	0.068	0.049	0.734	0.158	31.989	0.000	35.347	37.435	27.842	9.993
67	Vegetables	Canned or other vegetable products	0.104	0.095	0.812	0.084	15.632	0.001	10.088	18.978	31.239	12.697
68	Vegetables	Fruit vegetables	0.049	0.060	0.645	0.084	13.560	0.000	22.079	10.892	24.632	9.783
69	Vegetables	Leaf vegetables	0.050	0.083	0.624	0.068	59.160	0.000	9.295	58.486	39.813	22.500
70	Vegetables	Mushrooms	0.077	0.290	5.604	0.196	21.154	0.013	3.446	4.880	65.849	9.365
71	Vegetables	Onion-family vegetables	0.032	0.020	0.437	0.153	13.305	0.000	10.339	32.693	42.315	10.939
72	Vegetables	Root vegetables and tubers	0.083	0.081	1.028	0.092	44.490	0.000	20.470	29.726	47.055	15.344
73	water & drinks	Beer, cider and long drink	0.009	0.019	0.327	0.016	5.483	0.000	0.275	4.709	18.586	9.052
74	water & drinks	Coffee beverages	0.000	0.005	0.677	0.000	0.058	0.003	0.008	4.578	8.873	10.636
75	water & drinks	Juice	0.064	0.027	0.888	0.092	20.237	0.000	30.836	13.415	15.377	9.783
76	water & drinks	Soft drinks (incl. sport drinks)	0.006	0.031	0.740	0.110	1.994	0.102	3.838	3.692	7.202	3.021
77	water & drinks	Tea	0.001	0.008	0.001	0.000	2.067	0.000	0.838	0.477	1.352	0.250
78	water & drinks	Water	0.000	0.000	0.000	0.000	0.000	0.000	0.000	3.023	0.001	0.100
79	water & drinks	Wines and spirits	0.004	0.018	0.140	0.035	0.548	0.000	0.000	6.653	7.227	8.506

Supplemental Data 2: data for different sustainability metrics of foods by category (male)

Male			Nutrient composition (per 100g)									
#	Food group	Food category	Potassium (g)	Iron (mg)	Zinc (mg)	Iodine (µg)	Selenium (µg)	Copper (mg)	Sodium (mg)	NACL (g)	Saturated fatty acids (g)	Total fat (g)
	group	category	K	FE	ZN	ID	SE	CU	Sodium	NACL	fasatra	FAT
1	Dairy	Cheese, ripened or processed <=17 %	0.234	0.206	2.012	23.182	16.456	0.165	1030.204	2.625	6.758	10.914
2	Dairy	Cheese, ripened or processed > 17 %	0.083	0.271	3.738	26.889	19.125	0.359	515.081	1.312	19.108	29.642
3	Dairy	Cheese, unripened, fresh cheese <=15 %	0.100	0.120	1.350	17.000	12.218	0.034	300.000	0.764	1.371	2.000
4	Dairy	Cheese, unripened, fresh cheese >15 %	0.175	0.148	0.671	17.156	4.642	0.047	168.009	0.428	5.536	8.491
5	Dairy	Cream	0.117	0.156	0.353	12.822	2.567	0.010	102.235	0.260	15.081	23.169
6	Dairy	Dairy dessert	0.166	0.178	0.500	16.601	4.586	0.013	55.373	0.141	2.529	5.298
7	Dairy	Dairy imitates	0.182	0.892	0.365	3.526	1.499	0.115	78.811	0.201	1.084	4.269
8	Dairy	Fermented milk products, other	0.156	0.408	0.288	8.834	2.295	0.071	54.517	0.139	3.125	5.124
9	Dairy	Milk powder	1.710	0.450	4.738	149.941	19.994	0.099	197.593	0.503	3.002	4.764
10	Dairy	Milk, fat <=2%	0.151	0.050	0.410	14.725	2.750	0.009	41.000	0.104	1.003	1.438
11	Dairy	Milk, fat >2%	0.160	0.062	0.442	15.121	2.882	0.010	44.753	0.114	2.283	3.549
12	Dairy	Quark	0.170	0.050	0.550	17.000	3.853	0.026	99.140	0.253	0.126	0.400
13	Dairy	Skimmed milk	0.166	0.040	0.428	14.738	2.740	0.010	45.935	0.117	0.068	0.100
14	Dairy	Soured and cultured milks	0.135	0.389	0.457	12.687	2.442	0.024	36.683	0.093	0.145	0.668
15	Dairy	Yoghurt	0.171	0.074	0.428	14.743	2.620	0.013	44.216	0.113	0.766	1.227
16	Eggs	Eggs	0.131	1.726	1.420	50.480	33.388	0.051	235.302	0.600	2.893	11.705
17	Fats	Blended fat and oil	0.010	0.035	0.033	4.946	0.173	0.004	350.638	0.893	14.285	75.248
18	Fats	Butter, milk fat	0.018	0.180	0.080	3.167	0.500	0.011	589.035	1.501	52.829	81.391
19	Fats	Margarine and similar products	0.021	0.120	0.050	0.000	0.070	0.012	500.000	1.274	31.206	80.000
20	Fats	Salad dressings and mayonnaises	0.061	0.488	0.474	28.143	7.173	0.021	326.785	0.833	12.251	56.924
21	Fats	Vegetable fat and oil	0.000	0.002	0.000	0.000	0.000	0.000	0.000	0.000	11.334	100.000
22	Fish/Seafood	Crustaceans and molluscs	0.070	3.600	1.310	204.550	22.264	0.240	151.575	0.386	0.146	0.796
23	Fish/Seafood	Fish	0.355	0.398	0.636	53.494	23.713	0.055	47.832	0.122	1.561	7.881
24	Fish/Seafood	Fish products	0.330	0.736	0.978	29.299	31.007	0.068	613.438	1.563	1.482	10.053
25	Fruits	Berries	0.159	0.733	0.139	0.997	1.518	0.065	2.441	0.006	0.033	0.643
26	Fruits	Canned or dried fruit	0.179	0.483	0.164	0.961	0.203	0.087	2.218	0.006	2.185	15.539
27	Fruits	Citrus fruits	0.150	0.178	0.107	0.664	0.051	0.064	1.610	0.004	0.026	0.192
28	Fruits	Miscellaneous fruits	0.341	0.907	0.169	0.968	0.344	0.114	4.757	0.012	0.183	1.379
29	Fruits	Pome fruits	0.114	0.113	0.032	1.130	0.176	0.044	0.979	0.002	0.021	0.098
30	Grains	Breakfast cereal	0.092	0.811	0.585	13.306	1.918	0.086	154.870	0.395	0.249	1.223
31	Grains	Fine bakerly wares	0.149	0.676	0.674	16.420	7.722	0.087	186.288	0.475	5.132	12.121
32	Grains	Multigrain bread and rolls	0.278	2.154	1.746	17.800	4.422	0.267	432.946	1.103	0.491	3.148
33	Grains	Noodles	0.072	1.358	0.470	3.633	3.353	0.079	86.806	0.221	0.341	1.059
34	Grains	Other cereal products	0.365	2.170	1.930	9.916	105.046	0.630	382.086	0.974	4.383	27.265
35	Grains	Other grain and grain flour	0.273	2.829	1.932	9.199	6.084	0.320	1.972	0.005	0.410	2.726
36	Grains	Pasta	0.064	1.178	0.391	1.568	4.271	0.069	7.620	0.019	0.105	0.623
37	Grains	Rice and rice flour	0.085	0.815	0.604	1.671	5.061	0.109	2.603	0.007	0.160	0.756
38	Grains	Rye and rye flour	0.394	3.091	2.628	6.097	4.753	0.384	1.548	0.004	0.242	1.887
39	Grains	Rye bread and rolls	0.299	2.195	1.913	8.908	3.938	0.260	391.572	0.998	0.171	1.411
40	Grains	Starch	0.065	1.069	0.225	19.832	1.701	0.068	13.377	0.034	0.313	0.751
41	Grains	Wheat and wheat flour	0.164	0.988	0.900	9.956	9.955	0.181	1.072	0.003	0.209	1.590
42	Grains	Wheat bread and rolls	0.155	1.249	0.992	14.736	6.884	0.176	388.188	0.989	0.650	2.543
43	Legumes/Nuts	Legume products	0.705	3.891	2.384	4.428	6.821	0.843	139.016	0.354	3.471	24.656
44	Legumes/Nuts	Legumes	0.406	1.960	1.261	1.130	1.107	0.214	2.252	0.006	0.161	0.768
45	Legumes/Nuts	Nuts and seeds	0.676	4.372	4.029	6.047	7.983	1.359	6.114	0.016	6.314	47.200
46	Legumes/Nuts	Soya and soya products	1.232	7.047	2.793	3.370	9.700	1.137	136.447	0.348	0.881	7.783
47	Meats	Beef	0.449	3.441	6.822	5.712	23.017	0.112	97.133	0.247	2.713	6.247
48	Meats	Edible offal	0.323	10.978	3.800	12.721	62.433	2.153	585.458	1.492	3.963	11.013
49	Meats	Game	0.527	6.410	5.976	30.034	20.923	0.442	472.563	1.204	1.874	4.730
50	Meats	Lamb	0.382	2.871	4.414	28.620	16.107	0.121	463.960	1.182	6.811	14.909
51	Meats	Meat cuts	0.242	0.646	1.426	7.457	12.518	0.052	790.904	2.015	2.512	7.086
52	Meats	Pork	0.382	1.096	2.855	31.410	24.677	0.118	510.420	1.301	6.600	18.935
53	Meats	Poultry	0.210	1.387	1.535	17.282	15.309	0.069	69.313	0.177	3.370	13.778
54	Meats	Sausage	0.213	0.987	1.465	13.824	10.688	0.057	858.094	2.186	6.924	18.140
55	Meats	Mycoprotein	0.100	0.500	9.000	0.000	20.000	0.500	5.000	0.013	1.500	3.250
56	Spices/Condiments	Condiments	0.065	0.447	0.204	20.883	3.326	0.031	678.501	1.729	3.982	11.507
57	Spices/Condiments	Dried spices and herbs	0.627	4.304	1.013	4.439	2.622	0.158	24.538	0.063	0.099	0.706
58	Spices/Condiments	Miscellaneous ingredients	0.947	2.332	3.718	2.841	0.883	0.148	5508.065	14.035	2.844	6.369
59	Spices/Condiments	Salt	0.086	0.768	0.080	2293.086	0.460	0.179	38651.384	98.484	0.000	0.000
60	Starchy/Tubers	Potato products	1.014	1.525	0.677	0.403	3.204	0.188	486.157	1.239	3.890	26.314
61	Starchy/Tubers	Potatoes	0.500	0.670	0.310	1.000	0.637	0.091	1.000	0.003	0.025	0.114
62	Sugar	Chocolate	0.239	1.057	0.616	4.560	0.762	0.007	21.663	0.055	7.518	15.517
63	Sugar	Jams	0.106	0.302	0.107	2.274	0.378	0.043	1.472	0.004	0.042	0.239
64	Sugar	Non-chocolate confectionery	0.158	2.181	0.381	10.974	0.931	0.146	107.770	0.275	0.653	1.320
65	Sugar	Sugar and syrups	0.023	0.259	0.031	4.961	0.518	0.025	3.089	0.008	0.000	0.000
66	Vegetables	Cabbages	0.245	0.207	0.117	0.918	10.000	0.011	5.638	0.014	0.024	0.100
67	Vegetables	Canned or other vegetable products	0.267	0.526	0.187	13.095	0.800	0.077	811.556	2.068	0.210	0.670
68	Vegetables	Fruit vegetables	0.192	0.355	0.132	0.965	0.432	0.040	281.031	0.716	0.031	0.241
69	Vegetables	Leaf vegetables	0.373	0.750	0.492	1.012	0.788	0.051	21.385	0.054	0.026	0.217
70	Vegetables	Mushrooms	0.275	1.789	0.725	1.374	8.793	0.285	2.337	0.006	0.070	0.737
71	Vegetables	Onion-family vegetables	0.228	0.520	0.410	1.035	0.531	0.059	3.624	0.009	0.022	0.110
72	Vegetables	Root vegetables and tubers	0.378	0.495	0.377	1.054	3.651	0.055	20.241	0.052	0.040	0.234
73	water & drinks	Beer, cider and long drink	0.037	0.034	0.008	0.914	0.057	0.006	3.105	0.008	0.000	0.000
74	water & drinks	Coffee beverages	0.096	0.046	0.025	1.066	0.078	0.002	0.639	0.002	0.022	0.115
75	water & drinks	Juice	0.157	0.338	0.067	0.980	0.207	0.029	3.974	0.010	0.248	0.747
76	water & drinks	Soft drinks (incl. sport drinks)	0.036	0.038	0.010	0.613	0.086	0.007	4.932	0.013	0.002	0.046
77	water & drinks	Tea	0.012	0.024	0.010	1.248	0.081	0.005	0.260	0.001	0.000	0.000
78	water & drinks	Water	0.000	0.000	0.010	1.000	0.050	0.000	1.033	0.003	0.000	0.000
79	water & drinks	Wines and spirits	0.070	0.527	0.035	1.463	0.079	0.010	3.766	0.010	0.000	0.127

Male			Nutrient composition (per 100g)								
#	Food group	Food category	Carbohydrates (g)	Dietary fiber (g)	Proteins (g)	Alcohol (g)	Energy (kJ)	Cis-MUFA (g)	Cis-PUFA (g)	Alpha-linolenic acid (ALA) (g)	Linoleic acid + ALA (g)
	group	category	CHOAVL	FIBT	PROT	ALC	ENERC	famcira	fapura	ALA	ALALA
1	Dairy	Cheese, ripened or processed <=17 %	2.713	0.000	18.644	0.000	767.486	2.409	0.261	0.050	0.195
2	Dairy	Cheese, ripened or processed > 17 %	0.014	0.000	24.970	0.000	1529.492	7.026	0.769	0.137	0.554
3	Dairy	Cheese, unripened, fresh cheese <=15 %	2.500	0.000	15.810	0.000	385.270	0.479	0.056	0.012	0.038
4	Dairy	Cheese, unripened, fresh cheese >15 %	3.390	0.187	10.622	0.000	567.487	2.086	0.234	0.069	0.187
5	Dairy	Cream	3.339	0.023	1.742	0.048	940.944	5.960	0.842	0.134	0.556
6	Dairy	Dairy dessert	15.733	0.023	4.033	0.000	534.574	1.330	0.292	0.049	0.244
7	Dairy	Dairy imitates	4.663	1.060	5.262	0.000	336.046	1.553	1.192	0.163	0.729
8	Dairy	Fermented milk products, other	8.328	0.764	3.207	0.000	395.962	1.091	0.462	0.114	0.437
9	Dairy	Milk powder	19.859	0.000	64.473	0.000	1609.900	1.483	0.153	0.007	0.026
10	Dairy	Milk, fat <=2%	4.125	0.000	3.373	0.000	183.245	0.343	0.032	0.005	0.025
11	Dairy	Milk, fat >2%	4.883	0.001	3.004	0.000	268.012	0.750	0.077	0.012	0.057
12	Dairy	Quark	3.100	0.000	9.810	0.000	244.710	0.054	0.014	0.001	0.007
13	Dairy	Skimmed milk	4.250	0.000	3.698	0.000	141.394	0.021	0.004	0.000	0.003
14	Dairy	Soured and cultured milks	6.241	0.234	2.990	0.000	192.499	0.297	0.189	0.036	0.188
15	Dairy	Yoghurt	8.818	0.098	3.152	0.000	255.525	0.253	0.031	0.006	0.025
16	Eggs	Eggs	0.308	0.000	12.723	0.000	654.622	4.768	1.964	0.243	1.678
17	Fats	Blended fat and oil	0.324	0.000	0.375	0.000	2800.220	36.737	19.472	6.131	19.240
18	Fats	Butter, milk fat	0.796	0.000	1.188	0.000	3045.153	19.422	2.573	0.361	1.403
19	Fats	Margarine and similar products	0.300	0.000	0.380	0.000	2971.480	28.046	12.491	2.368	12.491
20	Fats	Salad dressings and mayonnaises	3.682	0.063	1.848	0.000	2204.308	27.068	14.862	4.036	14.693
21	Fats	Vegetable fat and oil	0.000	0.000	0.000	0.000	3699.988	61.355	23.033	6.025	22.692
22	Fish/Seafood	Crustaceans and molluscs	0.000	0.000	14.448	0.000	275.014	0.167	0.282	0.005	0.015
23	Fish/Seafood	Fish	0.000	0.000	16.944	0.000	579.634	2.672	2.727	0.137	0.438
24	Fish/Seafood	Fish products	2.215	0.105	19.556	0.000	743.024	4.257	3.179	0.494	2.110
25	Fruits	Berries	10.728	2.753	0.705	0.000	256.028	0.036	0.153	0.072	0.152
26	Fruits	Canned or dried fruit	15.790	1.848	0.741	0.000	877.968	10.484	1.750	0.111	1.747
27	Fruits	Citrus fruits	8.657	2.237	0.562	0.000	189.688	0.029	0.058	0.027	0.058
28	Fruits	Miscellaneous fruits	16.183	1.837	1.229	0.000	370.157	0.669	0.144	0.040	0.143
29	Fruits	Pome fruits	8.269	1.933	0.206	0.000	171.088	0.006	0.049	0.010	0.048
30	Grains	Breakfast cereal	11.651	1.592	2.401	0.000	300.392	0.402	0.444	0.097	0.443
31	Grains	Fine bakerly wares	42.370	1.691	6.744	0.000	1298.152	3.708	1.493	0.267	1.423
32	Grains	Multigrain bread and rolls	38.930	7.988	7.998	0.000	981.118	0.904	1.329	0.307	1.326
33	Grains	Noodles	25.878	1.447	3.900	0.000	557.395	0.268	0.279	0.016	0.279
34	Grains	Other cereal products	45.270	5.114	12.263	0.000	2035.982	11.948	8.289	0.838	8.286
35	Grains	Other grain and grain flour	38.960	4.793	7.448	0.000	936.690	0.754	1.194	0.077	1.186
36	Grains	Pasta	20.366	1.440	3.064	0.000	432.982	0.164	0.240	0.012	0.239
37	Grains	Rice and rice flour	25.425	1.666	2.702	0.000	519.897	0.254	0.260	0.011	0.260
38	Grains	Rye and rye flour	60.048	11.042	10.027	0.000	1360.052	0.264	0.910	0.115	0.910
39	Grains	Rye bread and rolls	44.350	10.035	7.466	0.000	1019.206	0.191	0.638	0.076	0.631
40	Grains	Starch	82.852	1.043	0.998	0.000	1461.075	0.138	0.066	0.003	0.066
41	Grains	Wheat and wheat flour	73.608	3.573	11.647	0.000	1537.954	0.166	0.689	0.040	0.689
42	Grains	Wheat bread and rolls	49.287	3.260	8.688	0.000	1106.044	0.658	0.773	0.088	0.772
43	Legumes/Nuts	Legume products	12.616	9.102	20.895	0.000	1553.770	9.568	7.507	0.103	7.507
44	Legumes/Nuts	Legumes	9.627	3.335	5.528	0.000	317.058	0.081	0.437	0.090	0.436
45	Legumes/Nuts	Nuts and seeds	12.144	7.837	22.554	0.000	2402.263	18.661	15.995	2.272	15.887
46	Legumes/Nuts	Soya and soya products	11.432	10.449	29.470	0.000	1044.079	2.772	3.553	0.607	3.510
47	Meats	Beef	0.000	0.000	29.745	0.000	736.817	2.380	0.311	0.042	0.211
48	Meats	Edible offal	6.329	1.227	17.410	0.000	821.349	3.973	1.775	0.122	1.299
49	Meats	Game	0.000	0.000	27.153	0.000	636.591	1.310	0.397	0.058	0.268
50	Meats	Lamb	0.000	0.000	22.740	0.000	938.280	5.587	0.712	0.134	0.432
51	Meats	Meat cuts	0.662	0.000	17.375	0.000	568.839	3.165	0.880	0.082	0.784
52	Meats	Pork	0.000	0.000	26.670	0.000	1154.040	8.487	2.605	0.188	2.253
53	Meats	Poultry	0.000	0.000	23.644	0.000	911.683	6.984	2.718	0.162	2.625
54	Meats	Sausage	4.438	0.072	12.508	0.000	1006.085	8.008	2.196	0.184	1.989
55	Meats	Mycoprotein	3.000	6.250	11.250	0.000	355.640	1.400	1.541	4.900	9.200
56	Spices/Condiments	Condiments	6.806	0.380	1.674	0.000	575.170	4.471	2.379	0.607	2.332
57	Spices/Condiments	Dried spices and herbs	2.587	2.694	2.583	0.000	164.116	0.079	0.252	0.161	0.251
58	Spices/Condiments	Miscellaneous ingredients	5.560	5.065	12.506	0.000	583.180	2.372	0.542	0.012	0.541
59	Spices/Condiments	Salt	0.000	0.000	0.576	0.000	9.807	0.000	0.000	0.000	0.000
60	Starchy/Tubers	Potato products	40.121	3.949	4.507	0.000	1782.770	9.657	9.235	1.592	9.232
61	Starchy/Tubers	Potatoes	15.500	1.400	1.880	0.000	315.350	0.002	0.064	0.029	0.064
62	Sugar	Chocolate	74.479	2.452	1.998	0.000	1891.267	5.342	1.189	0.144	1.186
63	Sugar	Jams	39.249	1.280	0.495	0.000	693.201	0.031	0.068	0.031	0.068
64	Sugar	Non-chocolate confectionery	83.247	0.853	1.347	0.000	1508.220	0.412	0.065	0.004	0.065
65	Sugar	Sugar and syrups	95.459	0.000	0.108	0.000	1624.644	0.000	0.000	0.000	0.000
66	Vegetables	Cabbages	4.673	1.932	1.208	0.000	125.232	0.008	0.079	0.050	0.076
67	Vegetables	Canned or other vegetable products	7.519	1.008	0.986	0.000	188.319	0.175	0.114	0.015	0.114
68	Vegetables	Fruit vegetables	3.406	0.883	0.713	0.000	93.553	0.022	0.082	0.016	0.082
69	Vegetables	Leaf vegetables	0.991	1.219	1.188	0.000	56.560	0.008	0.103	0.068	0.100
70	Vegetables	Mushrooms	2.637	2.009	2.618	0.000	139.757	0.013	0.158	0.000	0.152
71	Vegetables	Onion-family vegetables	5.036	1.200	1.386	0.000	129.645	0.016	0.053	0.004	0.053
72	Vegetables	Root vegetables and tubers	5.168	2.382	1.324	0.000	144.508	0.011	0.129	0.037	0.126
73	water & drinks	Beer, cider and long drink	3.595	0.001	0.334	3.489	168.951	0.000	0.000	0.000	0.000
74	water & drinks	Coffee beverages	0.405	0.011	0.328	0.000	16.827	0.008	0.001	0.000	0.001
75	water & drinks	Juice	10.332	0.169	0.652	0.000	222.926	0.272	0.145	0.026	0.144
76	water & drinks	Soft drinks (incl. sport drinks)	6.602	0.008	0.175	0.000	117.301	0.002	0.005	0.002	0.005
77	water & drinks	Tea	5.452	0.000	0.053	0.000	93.958	0.000	0.000	0.000	0.000
78	water & drinks	Water	0.054	0.000	0.000	0.000	0.916	0.000	0.000	0.000	0.000
79	water & drinks	Wines and spirits	1.659	0.000	0.245	13.623	439.852	0.000	0.000	0.000	0.000

Male			Nutrient composition (per 100g)							
**#**	**Food group**	**Food category**	**Docosahexaenoic acid (mg)**	**Histidine (mg)**	**Isoleucine (mg)**	**Leucine (mg)**	**Lysine (mg)**	**Methionine + cysteine (mg)**	**Phenylalanine + tyrosine (mg)**	**Threonine (mg)**
	group	category	DHA	HIS	ILE	LEU	LYS	met_cys	tyr_phe	THR
1	Dairy	Cheese, ripened or processed <=17 %	0.000	736.927	1029.781	1895.137	1910.482	658.494	2135.298	764.089
2	Dairy	Cheese, ripened or processed > 17 %	0.000	763.944	1133.563	2122.977	1913.995	775.065	2346.881	861.256
3	Dairy	Cheese, unripened, fresh cheese <=15 %	0.000	326.000	591.000	1116.000	934.000	335.000	1181.000	500.000
4	Dairy	Cheese, unripened, fresh cheese >15 %	0.685	205.754	370.331	696.224	555.410	240.500	707.886	300.995
5	Dairy	Cream	4.726	118.814	214.834	392.407	332.936	130.406	386.701	168.373
6	Dairy	Dairy dessert	5.411	104.936	228.203	383.855	312.093	137.780	372.652	174.390
7	Dairy	Dairy imitates	0.000	109.806	203.945	326.630	249.396	90.441	369.621	161.463
8	Dairy	Fermented milk products, other	0.000	108.955	205.328	377.478	334.910	135.657	401.582	163.388
9	Dairy	Milk powder	0.000	397.035	1028.845	1682.322	1414.584	648.376	1357.920	978.882
10	Dairy	Milk, fat <=2%	0.000	100.000	171.000	313.000	276.000	107.000	338.000	141.000
11	Dairy	Milk, fat >2%	0.000	95.191	163.305	299.534	264.458	102.191	322.611	134.267
12	Dairy	Quark	0.000	326.000	591.000	1116.000	934.000	335.000	1181.000	500.000
13	Dairy	Skimmed milk	0.000	102.000	174.000	319.000	282.000	109.000	345.000	144.000
14	Dairy	Soured and cultured milks	0.000	125.748	233.564	429.089	380.300	152.690	457.466	186.948
15	Dairy	Yoghurt	0.000	175.461	346.010	637.979	555.333	225.667	646.842	277.681
16	Eggs	Eggs	108.069	282.119	625.602	1000.664	834.236	607.884	1091.551	530.695
17	Fats	Blended fat and oil	0.000	23.000	52.000	84.000	68.000	29.000	82.000	39.000
18	Fats	Butter, milk fat	0.000	22.915	50.809	82.686	66.747	28.893	81.695	37.859
19	Fats	Margarine and similar products	0.000	23.000	52.000	84.000	68.000	29.000	82.000	39.000
20	Fats	Salad dressings and mayonnaises	19.433	16.974	37.419	54.883	44.419	31.422	62.883	34.440
21	Fats	Vegetable fat and oil	0.000	0.000	0.000	0.000	0.000	0.000	0.000	0.000
22	Fish/Seafood	Crustaceans and molluscs	118.106	302.000	718.000	1176.000	1290.000	584.000	1119.000	599.000
23	Fish/Seafood	Fish	1183.530	550.843	932.057	1598.526	1832.715	817.784	1509.930	859.510
24	Fish/Seafood	Fish products	476.148	676.897	1081.038	1914.343	2138.710	1030.498	1721.933	1026.588
25	Fruits	Berries	0.000	11.482	19.846	37.249	19.997	15.932	45.991	19.749
26	Fruits	Canned or dried fruit	0.000	7.776	8.967	17.371	19.770	8.722	20.503	10.853
27	Fruits	Citrus fruits	0.000	12.296	18.150	20.332	33.774	16.425	35.053	12.612
28	Fruits	Miscellaneous fruits	0.000	67.344	30.855	67.991	53.210	21.182	60.987	30.149
29	Fruits	Pome fruits	0.000	4.601	6.665	14.665	13.532	2.266	8.665	6.665
30	Grains	Breakfast cereal	0.000	197.953	344.497	552.015	285.816	309.448	645.189	323.124
31	Grains	Fine bakery wares	8.530	134.938	253.427	444.836	249.114	232.943	483.172	202.276
32	Grains	Multigrain bread and rolls	0.000	205.094	334.685	576.031	299.433	325.267	645.535	280.598
33	Grains	Noodles	0.023	81.145	148.711	263.414	94.289	159.115	286.205	109.815
34	Grains	Other cereal products	0.085	338.544	537.706	1056.540	472.076	444.955	1147.469	442.974
35	Grains	Other grain and grain flour	6.655	308.723	483.026	990.765	560.256	504.714	1100.102	415.368
36	Grains	Pasta	0.094	285.101	481.687	956.869	286.175	387.435	873.007	436.643
37	Grains	Rice and rice flour	0.000	63.007	116.010	222.021	97.009	118.010	234.031	96.009
38	Grains	Rye and rye flour	0.000	246.704	373.543	849.634	387.471	416.357	892.777	489.126
39	Grains	Rye bread and rolls	0.000	183.047	320.862	582.375	234.338	313.804	627.608	256.513
40	Grains	Starch	0.000	213.633	359.446	610.802	511.371	215.207	680.393	320.694
41	Grains	Wheat and wheat flour	0.000	231.178	358.498	711.855	232.574	402.781	832.584	283.383
42	Grains	Wheat bread and rolls	0.000	189.452	307.214	560.770	243.880	313.717	630.206	258.809
43	Legumes/Nuts	Legume products	0.000	475.572	804.391	1380.675	1071.098	537.790	1508.853	733.260
44	Legumes/Nuts	Legumes	0.000	150.659	255.298	436.089	408.021	148.242	439.369	249.657
45	Legumes/Nuts	Nuts and seeds	0.000	556.455	855.461	1536.033	878.765	671.690	1894.805	783.727
46	Legumes/Nuts	Soya and soya products	0.000	449.454	858.878	1400.241	894.466	329.149	1533.168	783.046
47	Meats	Beef	0.000	870.188	1026.406	1854.356	1948.272	1106.000	1782.012	902.544
48	Meats	Edible offal	65.039	662.390	705.315	1456.387	1406.402	439.880	1316.606	721.700
49	Meats	Game	0.000	565.000	1371.000	2258.000	2016.000	1049.000	1774.000	1290.000
50	Meats	Lamb	0.000	841.000	1339.000	2295.000	2452.000	1106.000	2201.000	1314.000
51	Meats	Meat cuts	2.970	683.316	790.976	1411.878	1504.594	644.671	1288.655	787.385
52	Meats	Pork	17.048	1026.000	1203.000	2061.000	2310.000	1008.000	1920.000	1173.000
53	Meats	Poultry	21.490	634.477	1075.851	1593.461	1772.802	881.794	1555.703	909.078
54	Meats	Sausage	3.746	437.346	622.642	1075.333	1191.141	506.210	996.923	609.236
55	Meats	Mycoprotein	0.000	390.000	570.000	950.000	910.000	230.000	540.000	610.000
56	Spices/Condiments	Condiments	0.421	36.263	58.218	97.246	85.017	53.159	99.779	62.915
57	Spices/Condiments	Dried spices and herbs	0.000	67.091	146.149	230.907	187.677	65.726	242.521	119.909
58	Spices/Condiments	Miscellaneous ingredients	0.000	161.661	350.256	494.160	506.599	209.333	553.532	322.873
59	Spices/Condiments	Salt	0.000	0.000	0.000	0.000	0.000	0.000	0.000	0.000
60	Starchy/Tubers	Potato products	0.000	210.180	306.262	748.320	304.277	284.023	716.483	293.992
61	Starchy/Tubers	Potatoes	0.000	35.000	66.000	98.000	107.000	56.000	129.000	67.000
62	Sugar	Chocolate	0.000	67.000	151.000	236.000	195.000	88.000	333.000	154.000
63	Sugar	Jams	0.000	10.143	14.713	23.132	24.477	10.225	33.703	15.026
64	Sugar	Non-chocolate confectionery	0.000	192.258	220.021	537.888	249.295	175.758	690.150	189.329
65	Sugar	Sugar and syrups	0.000	26.314	53.646	90.412	70.589	58.539	1024.856	45.584
66	Vegetables	Cabbages	0.000	21.969	33.222	43.509	46.345	22.831	51.755	35.431
67	Vegetables	Canned or other vegetable products	0.000	19.230	25.999	36.940	37.909	14.874	44.222	29.446
68	Vegetables	Fruit vegetables	0.000	16.731	28.519	47.114	42.121	20.883	54.335	29.318
69	Vegetables	Leaf vegetables	0.000	17.735	49.380	55.811	54.387	22.148	62.250	42.964
70	Vegetables	Mushrooms	0.000	53.247	77.676	125.264	105.301	42.711	132.173	105.301
71	Vegetables	Onion-family vegetables	0.000	16.028	18.158	30.797	43.793	8.765	43.609	23.786
72	Vegetables	Root vegetables and tubers	0.000	40.804	70.350	100.117	108.209	77.785	107.563	137.989
73	water & drinks	Beer, cider and long drink	0.000	0.000	0.000	0.000	0.000	0.000	0.000	0.000
74	water & drinks	Coffee beverages	0.000	3.211	5.080	10.482	4.951	5.090	10.385	2.836
75	water & drinks	Juice	0.000	2.984	6.040	12.232	9.249	7.204	11.428	7.189
76	water & drinks	Soft drinks (incl. sport drinks)	0.000	0.563	1.408	2.817	1.972	1.690	2.535	1.690
77	water & drinks	Tea	0.000	2.000	2.000	5.000	1.000	2.000	5.000	1.000
78	water & drinks	Water	0.000	0.000	0.000	0.000	0.000	0.000	0.000	0.000
79	water & drinks	Wines and spirits	0.000	1.608	3.598	5.820	4.719	2.032	5.756	2.688

Supplemental Data 2: data for different sustainability metrics of foods by category (male)

Male			Nutrient composition (per 100g)		
#	Food group	Food category	Tryptophan (mg)	Valine (mg)	Total essential amino acids (mg)
	group	category	TRP	VAL	ess_aa_sum
1	Dairy	Cheese, ripened or processed <=17 %	295.093	1350.347	9549.819
2	Dairy	Cheese, ripened or processed > 17 %	351.547	1485.506	10384.782
3	Dairy	Cheese, unripened, fresh cheese <=15 %	147.000	748.000	5208.000
4	Dairy	Cheese, unripened, fresh cheese >15 %	116.199	447.898	3233.156
5	Dairy	Cream	52.983	248.726	1824.927
6	Dairy	Dairy dessert	53.718	258.708	1805.034
7	Dairy	Dairy imitates	57.346	199.619	1576.148
8	Dairy	Fermented milk products, other	38.418	281.776	1822.881
9	Dairy	Milk powder	268.020	1064.124	7912.814
10	Dairy	Milk, fat <=2%	42.000	216.000	1517.000
11	Dairy	Milk, fat >2%	40.076	206.382	1449.672
12	Dairy	Quark	147.000	748.000	5208.000
13	Dairy	Skimmed milk	43.000	221.000	1548.000
14	Dairy	Soured and cultured milks	45.589	316.374	2072.165
15	Dairy	Yoghurt	76.584	452.830	3028.880
16	Eggs	Eggs	152.083	761.089	5163.129
17	Fats	Blended fat and oil	12.000	57.000	397.000
18	Fats	Butter, milk fat	11.955	56.786	391.525
19	Fats	Margarine and similar products	12.000	57.000	397.000
20	Fats	Salad dressings and mayonnaises	9.979	41.906	294.912
21	Fats	Vegetable fat and oil	0.000	0.000	0.000
22	Fish/Seafood	Crustaceans and molluscs	207.000	696.000	6031.000
23	Fish/Seafood	Fish	215.550	1047.686	8443.452
24	Fish/Seafood	Fish products	262.351	1208.291	9933.599
25	Fruits	Berries	5.137	27.574	182.044
26	Fruits	Canned or dried fruit	4.374	14.128	102.610
27	Fruits	Citrus fruits	5.534	26.956	162.764
28	Fruits	Miscellaneous fruits	10.083	48.277	366.608
29	Fruits	Pome fruits	1.133	12.665	68.592
30	Grains	Breakfast cereal	97.683	424.958	2708.573
31	Grains	Fine bakery wares	75.031	289.467	2056.693
32	Grains	Multigrain bread and rolls	128.590	411.606	2780.233
33	Grains	Noodles	49.750	172.305	1162.889
34	Grains	Other cereal products	173.388	662.628	4576.629
35	Grains	Other grain and grain flour	202.213	641.132	4551.764
36	Grains	Pasta	170.768	558.765	3951.242
37	Grains	Rice and rice flour	31.003	164.014	996.091
38	Grains	Rye and rye flour	183.052	524.810	3872.617
39	Grains	Rye bread and rolls	96.524	381.211	2608.014
40	Grains	Starch	128.557	430.320	3121.099
41	Grains	Wheat and wheat flour	127.795	417.815	3066.826
42	Grains	Wheat bread and rolls	117.638	367.956	2570.943
43	Legumes/Nuts	Legume products	257.791	877.234	6792.684
44	Legumes/Nuts	Legumes	56.133	298.823	2233.263
45	Legumes/Nuts	Nuts and seeds	259.282	1065.683	7399.601
46	Legumes/Nuts	Soya and soya products	207.590	891.010	6534.359
47	Meats	Beef	258.676	1090.686	9701.925
48	Meats	Edible offal	195.107	975.534	7362.430
49	Meats	Game	403.000	1452.000	11022.000
50	Meats	Lamb	334.000	1440.000	11988.000
51	Meats	Meat cuts	178.339	867.601	7374.535
52	Meats	Pork	326.000	1394.000	11198.000
53	Meats	Poultry	241.816	1069.090	8731.283
54	Meats	Sausage	147.271	674.681	5651.085
55	Meats	Mycoprotein	180.000	600.000	4980.000
56	Spices/Condiments	Condiments	18.857	73.437	514.173
57	Spices/Condiments	Dried spices and herbs	39.745	178.935	1147.876
58	Spices/Condiments	Miscellaneous ingredients	80.214	379.592	2718.561
59	Spices/Condiments	Salt	0.000	0.000	0.000
60	Starchy/Tubers	Potato products	79.431	436.002	2961.412
61	Starchy/Tubers	Potatoes	21.000	103.000	610.000
62	Sugar	Chocolate	58.000	234.000	1322.000
63	Sugar	Jams	6.087	21.411	138.383
64	Sugar	Non-chocolate confectionery	79.354	279.445	2253.183
65	Sugar	Sugar and syrups	17.959	71.069	1392.209
66	Vegetables	Cabbages	11.108	43.057	278.633
67	Vegetables	Canned or other vegetable products	8.865	28.195	220.968
68	Vegetables	Fruit vegetables	7.783	33.259	249.835
69	Vegetables	Leaf vegetables	10.718	48.624	332.170
70	Vegetables	Mushrooms	28.234	201.361	810.451
71	Vegetables	Onion-family vegetables	15.065	26.531	205.910
72	Vegetables	Root vegetables and tubers	15.988	80.134	641.044
73	water & drinks	Beer, cider and long drink	0.000	0.000	0.000
74	water & drinks	Coffee beverages	0.719	6.733	41.163
75	water & drinks	Juice	1.145	9.597	51.721
76	water & drinks	Soft drinks (incl. sport drinks)	0.282	2.253	13.238
77	water & drinks	Tea	0.000	3.000	17.000
78	water & drinks	Water	0.000	0.000	0.000
79	water & drinks	Wines and spirits	0.846	3.979	27.617
